# *APOE4-APP* interactions exert early influences on cerebrovascular structure and function: implications for Alzheimer’s disease

**DOI:** 10.3389/fnins.2025.1629830

**Published:** 2025-10-24

**Authors:** Lanboling Guo, Jessica R. Gaunt, Calvin Chee Hoe Cheah, Albert I. Chen, Stephanie Claudine, Gavin S. Dawe, Eyleen Lay Keow Goh, Sok-Hong Kho, Pawan Kumar, Grace G. Y. Lim, Kah Leong Lim, Yun-An Lim, Takaomi C. Saido, Takashi Saito, Hiroki Sasaguri, Judy C. G. Sng, Yee Jie Yeap, Alaric K. K. Yip, Norliyana Zainolabidin, Toh Hean Ch’ng, George J. Augustine

**Affiliations:** ^1^Neuroscience and Mental Health Program, Lee Kong Chian School of Medicine, Nanyang Technological University, Singapore, Singapore; ^2^School of Biological Sciences, Nanyang Technological University, Singapore, Singapore; ^3^Center for Aging Research, Scintillon Institute, San Diego, CA, United States; ^4^Molecular Neurobiology Laboratory, Salk Institute, San Diego, CA, United States; ^5^Department of Pharmacology, Yong Loo Lin School of Medicine, National University of Singapore, Singapore, Singapore; ^6^Healthy Longevity Translational Research Programme, Yong Loo Lin School of Medicine, National University of Singapore, Singapore, Singapore; ^7^Precision Medicine Translational Research Programme, Yong Loo Lin School of Medicine, National University of Singapore, Singapore, Singapore; ^8^Neuroscience and Metabolic Phenotyping Core, National University of Singapore, Singapore, Singapore; ^9^Neurobiology Programme, Life Sciences Institute, National University of Singapore, Singapore, Singapore; ^10^RIKEN Center for Brain Science, Saitama, Japan; ^11^Department of Neurocognitive Science, Institute of Brain Science, Nagoya City University Graduate School of Medical Sciences, Nagoya, Japan; ^12^Dementia Pathophysiology Collaboration Unit, RIKEN Center for Brain Science, Wako, Japan; ^13^Temasek Life Sciences Laboratory, Singapore, Singapore

**Keywords:** *APOE4*, *APP*, dementia, transcriptomics, cerebral vasculature

## Abstract

**Background:**

*APOE4* and *APP* are two of the main genetic risk factors for Alzheimer’s disease (AD). Although there have been suggestions that these two factors interact, most of the *in vivo* evidence for such interactions comes from transgenic mouse models that suffer from complications associated with protein overexpression. Our goal was to examine the consequences of interactions between *APOE4* and *APP* on brain function while avoiding the use of transgenic mice.

**Methods:**

We generated and characterized double-mutant knock-in mice incorporating familial *APP* mutations and humanized *APOE4*.

**Results:**

In the brains of 3-month-old double-mutant mice there were significant alterations in vascular remodeling genes, vascular structure and blood–brain barrier permeability. These changes were not observed in either *APOE4* or *APP* single-mutant mice and, thus, were caused by interactions between the two genes. These interaction effects were transient, because they were absent in 8-month-old double-mutant mice.

**Conclusion:**

These findings indicate that early vascular changes, driven by the interaction of *APP* and *APOE4*, may influence the progression of AD. Our work highlights the need to focus on the synergistic vascular actions of *APOE4* and *APP*, particularly at younger ages.

## Introduction

One of the major hallmarks of Alzheimer’s disease (AD) is the deposition of amyloid plaques. These plaques are composed of proteolytic fragments of the amyloid precursor protein (APP; Haass et al., 2011), a transmembrane protein whose physiological functions include cell adhesion and signaling (Müller and Zheng, 2012). Cleavage of APP by *β*- and *γ*-secretases produces amyloid β (Aβ) fragments, which can oligomerize and form plaques (Haass et al., 2011; Chen et al., 2017). Mutations in the human genes for APP or the APP-directed secretases are among the highest genetic risk factors for familial AD (Querfurth and LaFerla, 2010; Tanzi, 2012; Bellenguez et al., 2022). As a result, a large number of AD therapeutic strategies have focused on APP or Aβ (Karran and De Strooper, 2022; Zhang et al., 2023), with the recent success of anti-amyloid monoclonal antibodies hinting at the potential of this approach (Budd Haeberlein et al., 2022; van Dyck et al., 2023; Sims et al., 2023).

A second important type of AD risk factor is the apolipoprotein ε4 (*APOE4*) allele. *APOE4* is the strongest genetic risk factor for sporadic, late-onset AD, with a 3-5-fold higher risk of AD in people with one copy of *APOE4* and a more than 10-fold higher risk for people with 2 copies of the gene (Corder et al., 1993; Farrer et al., 1997; Mahley and Huang, 2012; Neu et al., 2017; Belloy et al., 2019; Fortea et al., 2024). *APOE4* is a lipid carrier protein involved in transport of cholesterol and phospholipids. While the mechanisms linking *APOE4* to AD are unclear, it is likely that *APOE4* increases the risk of AD by promoting neurotoxicity and/or being less neuroprotective than proteins produced by the other *APOE* alleles*, APOE2* or *APOE3* (Corder et al., 1994; Kim et al., 2009; Liu et al., 2013; Raulin et al., 2022). Negative consequences of *APOE4* expression include adverse effects on neuronal synapses, cytoskeleton, and mitochondria (Mahley and Huang, 2012), as well as neurodegeneration (Shi et al., 2017; Montagne et al., 2021). *APOE4* also affects brain vasculature and impairs blood–brain barrier function (Hafezi-Moghadam et al., 2007; Halliday et al., 2016; Nation et al., 2019; Montagne et al., 2021; Liu et al., 2022). For these reasons, *APOE4* is also a promising therapeutic target for sporadic AD (Safieh et al., 2019).

Interactions between *APP* and *APOE4* may also be important in the etiology of AD. Nearly 30% of a Chinese cohort of familial AD subjects were reported to carry at least one *APOE4* allele, including approximately 14% of subjects with mutations in *APP* or in genes that encode components of *γ*-secretase, termed *PSEN* (Jia et al., 2020). While the influence of *APOE4* on familial AD symptoms is unclear (Bussy et al., 2019; Talboom et al., 2019; Almkvist et al., 2022), some reports indicate that *APOE4* enhances these symptoms (Bloss et al., 2008; Almkvist et al., 2022; Jia et al., 2020). *APP* and *APOE4* proteins potentially interact via a variety of mechanisms. *APOE4* binds to both Aβ and to plaques (Strittmatter et al., 1993; Holtzman, 2001; Wisniewski and Drummond, 2020), while *APP* binds to *APOE4* receptors (Hoe and Rebeck, 2008; Verghese et al., 2013). *APOE4* affects clearance of Aβ from the brain, potentially accelerating production of plaques (Verghese et al., 2013; Kanekiyo et al., 2014). Astrocytic expression of an *APOE4* receptor – the low-density LDL receptor-related protein 1 - causes Aβ aggregation and plaque formation (Liu et al., 2017), while eliminating *APOE4* from astrocytes enhances cerebrovascular protection (Xiong et al., 2023). Further, *APOE4* secretion from glial cells enhances APP transcription and Aβ secretion in differentiated human neurons (Huang et al., 2017). Such findings suggest that *APOE4/APP* interactions are also a potential target for AD therapeutics (Pankiewicz et al., 2014; Wisniewski and Drummond, 2020; Sawmiller et al., 2023).

To determine the physiological impact of interactions between *APP* and *APOE4*, mouse genetic models have been generated that express both *APOE4* and various forms of *APP* and/or *PSEN1* (Van Dooren et al., 2006; Montagne et al., 2021; Balu et al., 2023). While these studies have indicated numerous possible adverse consequences of *APP/APOE4* interactions, they are limited by their reliance on transgenic overexpression of *APP* and/or *PSEN1.* Overexpression yields numerous complications that hinder interpretation of results from these models (Watamura et al., 2022; Sasaguri et al., 2022; Zhong et al., 2024). To circumvent such problems, we have developed a new genetic model of AD based on a second-generation APP knock-in mouse that avoids artifacts associated with transgenic overexpression and has been widely used to determine how Aβ pathology alters brain function (Saito et al., 2014; Sasaguri et al., 2017). Crossing these mice with *APOE4* knock-in mice (Foley et al., 2022) produced a better model for studying *APP-APOE4* interactions. With this new mouse model, we observed novel, early and transitory changes in vascular remodeling genes that are associated with attendant changes in the structure and permeability of cerebral vasculature distinct from the effects of *APP* mutations or *APOE4* alone. To our knowledge, such early cerebrovascular changes have never been reported in any AD-APOE4 mouse model. Our results reveal that cerebral vascular alterations, driven by early interactions between *APOE4* and *APP,* could play a novel role in the early progression of some forms of AD.

## Methods

Mouse strains. WT mice were the C57BL/6JInv strain (The Jackson Laboratory, stock #000664), APP-TKI mice were *App^NL-G-F^* mice (C57BL/6-App<tm3(NL-G-F)Tcs>; RIKEN BioResource Research Center) and ApoE4 mice were Apoe^tm1.1(APOE*4)Adiuj^ (The Jackson Laboratory, stock #027894). DM mice were created by mating APP-TKI and *APOE4* mice and were homozygous for the *App* mutations and heterozygous for *APOE4*. Both male and female mice were examined, as indicated below. All mouse procedures performed were approved by the Institutional Animal Care and Use Committees of NTU, NUS and TLL.

Histology. Mice were transcardially perfused with phosphate-buffered saline (PBS), followed by ice-cold 4% paraformaldehyde (PFA) in PBS. Brains collected for immunohistochemical analysis were post-fixed in 4% PFA for 4 h at 4 °C, washed in PBS, then cryoprotected in 30% sucrose in 0.1 M phosphate buffer overnight. After sinking in 30% sucrose solution, brains were embedded in embedding medium (Tissue-Tek® OCT compound; Sakura) on dry ice, then stored at −80 °C. 30 μm coronal sections were cut using a Cryostat (CM1950; Leica) at −20 °C, and stored in cryoprotectant (50% 0.1 M PB, 30% ethylene glycol, 20% glycerol). Sections were washed in PBS and mounted on glass slides.

For immunohistochemical processing, tissue sections were permeabilized in PBS with 0.2% Triton X for four 5-min washes. Tissue sections were then incubated in primary antibody, in 2% horse serum in PBS with 0.1% Triton-X, at 4 °C for 48 h. Primary antibodies used in histological experiments were: mouse anti-Amyloid-Beta 6E10 (803,001; BioLegend; 1:1000), rat anti-CD68 (MCA1957GA; Bio-Rad; 1:1000), rabbit anti-Iba1 (0190197418; Fujifilm Wako; 1:1000). Following incubation, sections were washed for four 5-min washes in PBS with 0.2% Triton-X, and incubated for 24 h in secondary antibody and DAPI (1:1000), in 2% horse serum in PBS with 0.1% Triton-X, at 4 °C. The following secondary antibodies were used: Donkey anti-rat Alexa Fluor 488 (A-21202; Invitrogen; 1:1000), donkey anti-rabbit Alexa Fluor 555 (A-31572; Invitrogen; 1:1000), donkey anti-mouse Alexa Fluor 647 (A-31571; Invitrogen; 1:1000). Following incubation, sections were washed for four 5-min washes in PBS. Sections were covered with coverslips with ProLong™ Gold antifade mountant (P36930; Invitrogen).

Whole slides were imaged using a slide scanner (Axio Scan.Z1; Zeiss). Images were processed using ImageJ (NIH; USA), with 8 areas of interest measured within the hippocampus of 2 separate sections for each individual mouse. Microglia were identified using colocalization of Iba1-positive and DAPI-positive staining. Data analysis was performed using R programming language (R Core Team, 2020). Data are expressed as mean ± standard error of the mean (SEM). Two-way analysis of variance was used to calculate statistical significance. *Post hoc* testing was carried out using Tukey’s multiple comparison test.

Biochemistry. Three brains from each of WT, APP-TKI, DM, and *APOE4* male mice were cut into smaller pieces and lysed in ice-cold lysis buffer (50 mM Tris pH7.5, 1% Triton X-100 supplemented with 1% protease inhibitor cocktail). Hippocampal tissue was homogenized using a handheld TissuRuptor (Qiagen) equipped with a sterile, single-use saw-tooth probe, following the manufacturer’s guidelines. Briefly, homogenization was performed on ice at medium speed in short bursts (5–10 s) for a total of 30 s. Lysed samples were then ultracentrifuged at 100,000 x *g* for 1 h at 4 °C. The supernatant containing the soluble fraction was collected and stored at −80 °C.

For western blotting, protein extracts were quantified using the bicinchoninic acid assay (23,225, Thermo Scientific). The soluble fraction was resolved using SDS-PAGE with a 10% resolving gel and the proteins were transferred onto a 0.2 μM nitrocellulose membrane (1,620,112, Bio-Rad Laboratories) in transfer buffer (BUF-2020-10X4L, 1^st^ BASE) supplemented with 10% methanol for 1 h at room temperature at 100 V. Membranes were blocked using 5% non-fat dry milk (1,706,404, Bio-Rad Laboratories) and probed using an APP primary antibody (2452S, Cell Signaling Technology, 1:1000). ꞵ-actin was used as an endogenous loading control (A5441, Sigma-Aldrich). Antibodies were incubated overnight at 4 °C with gentle agitation, washed (x3) with TBS-T, and then incubated with anti-rabbit (ab97051, Abcam, 1:10000) HRP-conjugated antibodies for 50 min at room temperature in the dark. For the detection of bands, chemiluminescent (ECL) reagent (K-12043-D20, Advansta) was used. The band intensities were measured using ImageJ. Three independent technical replicates were performed for each blot.

To measure levels of RNA encoding APP, brains were removed, micro-dissected and flash-frozen in liquid nitrogen. Total RNA was extracted (using RNeasy Plus Mini kit, Qiagen) from tissue stored in Trizol and quantitative RT-PCR was performed to determine the mRNA levels of APP expressed in the hippocampus. The primers, which recognize both mouse and human APP, were: forward primer 5’ TCCGTGTGATCTACGAGCGCAT 3′ and reverse primer 5’ GCCAAGACATCGTCGGAGTAGT 3′ with PowerUp™ SYBR™ Green Master Mix (ThermoFisher Scientific). GAPDH was used as the endogenous reference gene with the following primers: forward primer 5′ AGG TCG GTG TGA ACG GAT TTG 3′ and reverse primer 5′ TGT AGA CCA TGT AGT TGA GGT CA 3′. For immunoblotting, tissue was lysed in RIPA buffer (Pierce, ThermoFisher Scientific) supplemented with a protease inhibitor (cOmplete, Sigma-Aldrich) and phosphatase inhibitor (PhosSTOP, Roche, Sigma-Aldrich). The protein concentration was measured using a BCA kit (Pierce, Thermo Scientific) and 50 ug of the lysate with 0.1 M DTT was subjected to SDS-PAGE followed by immunoblotting. APP levels were measured with the 22C11 antibody (14–9,749-82, Invitrogen), phospho-Tau (Ser396) was measured with the PHF13 antibody (9,632, Cell Signaling Technology), phospho-Tau (Ser202, Thr205) was measured with the AT8 antibody (MN1020, Invitrogen) and total Tau was measured by the Tau-5 antibody (ab80579, Abcam). *β*-actin (13E5 clone, 4,970, Cell Signaling Technology) was used as the endogenous control for all blots. For the 3-month-old group total protein was extracted from hippocampal tissue stored in Trizol by precipitating the proteins in the phenol-ethanol solution supplemented with isopropanol and solubilizing the pellet in lysis buffer (20 mM EDTA, 140 mM NaCl, 5% SDS, 100 mM Tris pH 8.0, 50 mM NaF, 1 mM activated NaOv, protease inhibitor) while incubating at 50 °C for 5 h followed by overnight incubation at 37 °C (Kopec et al., 2017) after which BCA and immunoblotting was conducted in a similar manner as detailed above.

Transcriptomics. Whole hippocampi were dissected on ice from female mice aged 3 months and snap-frozen in TRIzol reagent (Invitrogen). We reasoned that female *ApoE4* carriers would be more likely to interact with the APP-TKI genotype, due to a female-specific increase in vulnerability to AD for the *APOE4* allele (Gamache et al., 2020) as well as an earlier onset of Aβ-based AD in females (Navakkode et al., 2021).

Samples were collected and sequenced in two batches: Batch 1 - WT, APP-TKI, and DM (*N =* 3 per group) and Batch 2 - WT and *APOE4* (*N =* 3 per group). Samples were homogenized in TRIzol, extracted using chloroform and 100% ethanol, and then column-purified. Directional polyA-enriched mRNA libraries were prepared and 150 bp paired-end reads were obtained by sequencing on the Illumina Novaseq 6,000 platform by NovogeneAIT Genomics (Singapore).

Initial processing steps were performed on the Gekko high-performance computing system (Nanyang Technological University, Singapore). First, adapters and low-quality bases were trimmed using Trimmomatic (v0.39; Bolger et al., 2014). STAR (v2.7.1a, Dobin et al., 2013) was then used to align paired-end reads to GENCODE mouse genome assembly release M24 supplemented with the human APOE RNA sequence obtained from Jackson laboratories. Reads per gene were quantified using HTseq (v0.11.2, Anders et al., 2015). Downstream analyses were conducted using R v4.0.2 (R Core Team, 2020) and Bioconductor (Gentleman et al., 2004). Counts for human and mouse APOE were combined, then data were filtered to retain genes with CPM > 1 in a minimum of three samples (16,171 genes) and then upper quartile normalised. Robust likelihood ratio tests for differential expression were conducted separately for each batch using edgeR (Robinson et al., 2010; McCarthy et al., 2012; Zhou et al., 2014) to compare each genotype to WT and APP-TKI to DM. A false discovery rate threshold of 10% was used to identify differentially expressed genes (DEGs) and non-protein-coding genes were removed. Hierarchical clustering was performed on log_2_ fold changes (LFCs) compared to WT for DEGs, using 1 - Pearson-correlation as the dissimilarity index and Ward’s minimum variance linkage method. The R packages gplots (Warnes et al., 2015) and ggplot2 (Wickham, 2016) were used for plotting figures. Gene Ontology analyses were conducted on DEGs compared to all expressed genes using topGO (Alexa et al., 2006) with the Fisher elimination method (significance threshold *p <* 0.01). Enrichment of APP-TKI vs. DM DEGs in the MGI Mammalian Phenotype database (Smith and Eppig, 2009) was analysed using the Enrichr and Enrichment Analysis Visualizer web tools (Chen et al., 2013; Xie et al., 2021). Data were further compared (250 protein-coding DEGs identified in our data) with publicly available datasets from Zhang et al. (2014) and Castillo et al. (2017). To determine cell-type enrichment of DEGs, z-scores were calculated for each gene from expression values for each cell type in the Zhang et al. (2014) database, measured in fragments per kilobase of exon per million mapped fragments (FPKM). FPKM values were averaged for cell types in the oligodendrocyte lineage. All sequencing datasets can be downloaded from the NCBI GEO repository (Accession number: GSE242751).

Quantitative PCR analysis was done using RNA extracted from the hippocampus of both male and female mice, which was then converted to cDNA using the iScript cDNA Synthesis Kit (Bio-Rad). After cDNA conversion, the relative amounts of mRNA from 7 genes - *Acta2, Anxa2, Cdh5, Flt1, Pecam1, Ptprb, Vwf* - were obtained using qPCR. The primers for these genes, as well as 2 housekeeping genes (*β-actin*, *Gapdh*), are listed in the Supplementary Table S1.

*In vivo* vascular imaging. Male mice, aged 3 months and 7–9 months, were anesthezed with 2% isoflurane, mounted on a stereotaxic frame, and anesthesia was maintained with 1–1.5% isoflurane. A 5.5 mm diameter cranial window was centered at ML, +1.5 mm, AP, −0.3 mm. Two coverslips (5.5 mm and 7.5 mm) were glued together with Norland optical adhesive 61. We glued the coverslips onto the cranial window with Metabond (Japan). 1.5% agarose was filled in the gap between the coverslip and the brain. A head plate was glued on top of the coverslips. Painkiller (Buprenorphine: 0.1 mg/kg), antibiotic (Baytril: 12 mg/kg), and anti-inflammatory (Dexamethasone: 0.2 mg/kg) were given. We waited 4 to 6 weeks after surgery, to allow ample time for recovery prior to imaging.

During imaging, mice were initially anesthetized with 1.5% isoflurane that was later reduced to 1%. Five minutes prior to imaging, 100 μL of a solution containing fluorescein isothiocyanate-conjugated 40 kDa dextran (Sigma-Aldrich, 50 mg/mL) and tetramethylrhodamine-conjugated 70 kDa dextran (ThermoFisher, 50 mg/mL) was delivered through retro-orbital injection. Vessels were imaged with a two-photon microscope (Ultima two-photon microscope, Bruker, USA). The fluorophore was excited (860 nm) with a Ti: Sapphire femtosecond laser (Coherent Chameleon). Images were taken for 1 h at 5-min intervals, using a water immersion objective (Nikon 16X/0.8 NA). Each z-stack contained five layers (512×512) collected at 1 μm intervals.

Fiji (ImageJ), MATLAB and Python were used to analyze vessel permeability and structure. A U-net-based framework was used to segment blood vessels (Khanal and Estrada, 2020). We calculated the maximal intensity of the 5-layer z-stack images. The average fluorescence intensity of the extravascular space was divided by that of the vessels to get the outside/inside (O/I) ratio. For structural analysis, the same z-stack images of superficial vessels were used. Instead of using the MATLAB function bwskel, the code of 2D Average Outward Flux Skeletons was modified to extract skeletons (Siddiqi et al., 2002; Dimitrov et al., 2003; Rezanejad and Siddiqi, 2013). Vessel diameter was then measured by using an Euclidean distance transform to find the shortest distance between the skeleton (central line) and vessel edge. The total length of the vascular skeleton, normalized by area, was measured to determine vascular density. The number of branch points per unit area was measured by finding the intersection of vessels on segmented images.

## Results

We investigated interactions between *APP* mutations and *APOE4* in the progression of AD pathophysiology by comparing mice across four different genotypes: *APP^NL-G-F/NL-G-F^*, which harbors three knock-in mutations in the humanized Aβ region of *APP* (referred to here as APP-TKI; Saito et al., 2014; Sasaguri et al., 2017); *ApoE4^+/wt^* mice with a single allele of human *APOE4* knocked-in (ApoE4; Foley et al., 2022); the double-mutant (DM) *APP^NL-G-F/NL-G-F^; ApoE4^+/wt^* mice produced by crossing these two lines; and wild-type (WT) mice, which served as controls. Our analyses largely focused on the hippocampus, a primary target of early stage AD (Rao et al., 2022; Hanseeuw et al., 2023).

### *APOE4* does not affect *App* levels

**Figure 1:**
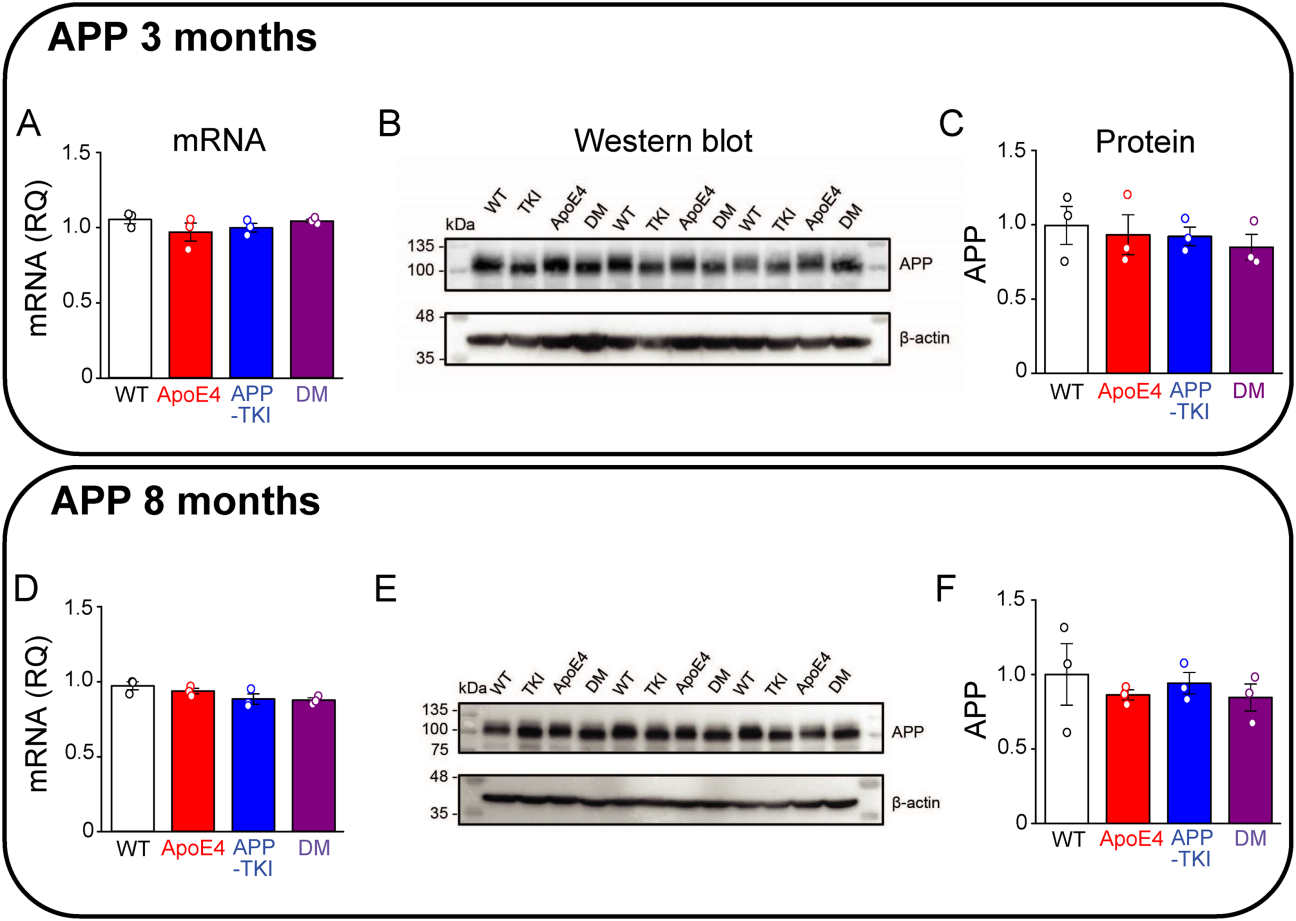
Co-expression of mutant APP and ApoE4 does not alter hippocampal APP expression. Measurements of APP in hippocampal tissue from 3-month-old **(A–C)** and 8-month-old **(D–F)** mice. **(A,D)** *APP* mRNA levels were determined by RT-qPCR, using *Gapdh* as an endogenous control, for each genotype (*N =* 3). Data are represented as RQ values, with points indicating means for three technical replicates for each mouse, bars showing mean values across mice (*N =* 3) and error bars indicating ± 1 SEM. One-way ANOVA showed no significant differences across the four genotypes for both age groups. **(B,E)** APP protein levels were determined in the FA-solubilized pellet fraction of hippocampal tissue by western blotting, with β-actin used as a loading control. **(C,F)** Semi-quantitative densitometric analysis of western blot APP bands, relative to β-actin. Points indicate means for individual mice, bars show mean values across mice (*N =* 3) and error bars indicate ± 1 SEM for each genotype group. No significant differences were detected between groups in one-way ANOVA.

Because *APP* is thought to be central to AD pathogenesis (O’Brien and Wong, 2011), we began by examining *APP* levels in the hippocampus of the various mouse lines. To investigate the influence of age, mice were examined at both a young age (3 months old) and an older age (8 months old). Both mRNA and protein levels of APP were similar across the hippocampus of all four genotypes - WT, ApoE4, APP-TKI, and DM - in 3-month-old mice (Figures 1A–C). Likewise, both mRNA and protein levels of APP were similar across all four genotypes in 8-month-old mice (Figures 1D–F). Similar results were obtained from measurements of APP mRNA and protein levels in cortical tissue as well (not shown).

**Figure 2:**
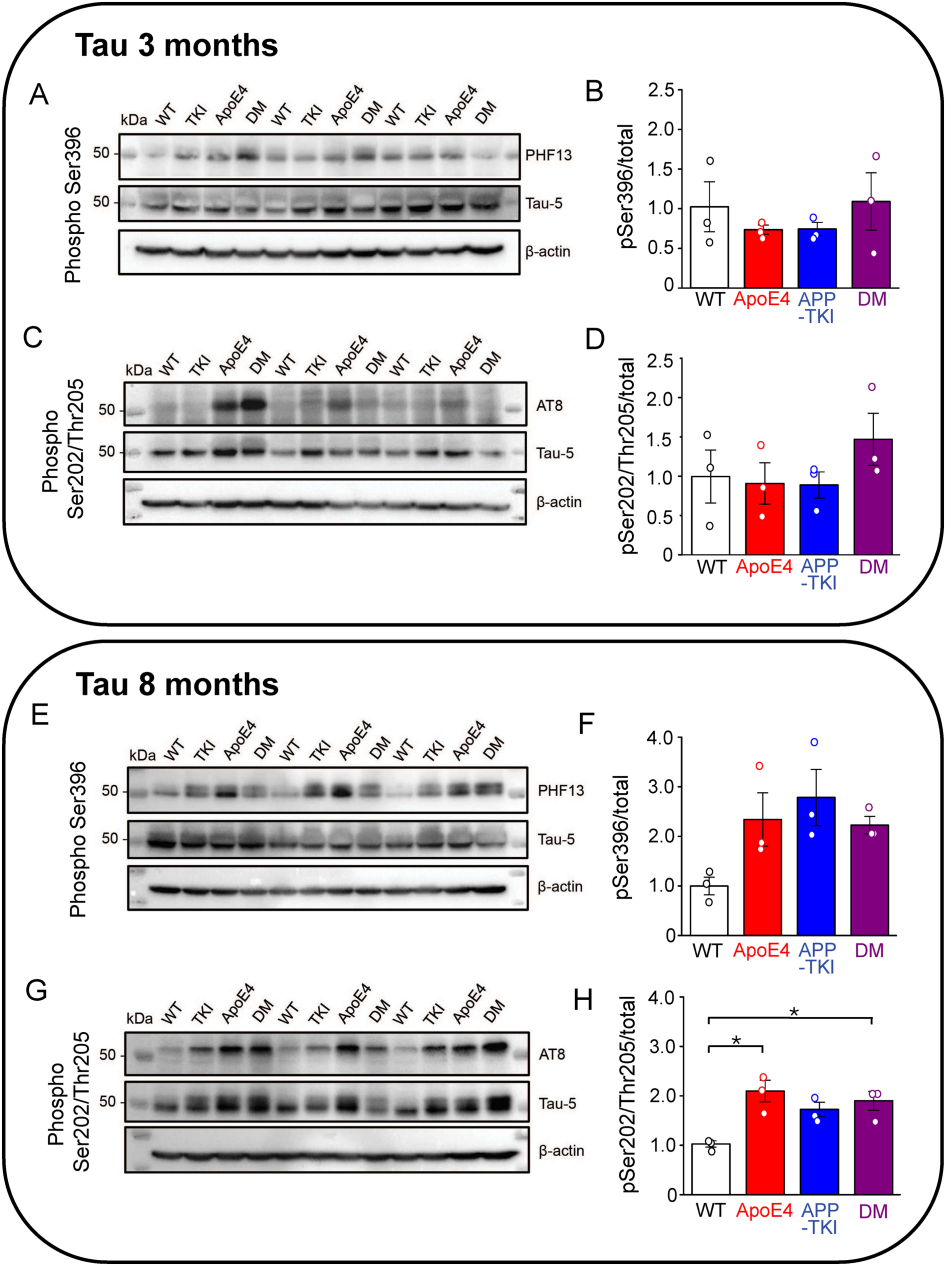
Phosphorylated hippocampal tau is higher in AD mutant mice at 8 months. Measurements of hippocampal phosphorylated tau from 3-month-old **(A–D)** and 8-month-old **(E–H)** mice. **(A,C,E,G)** Tau protein levels were determined in the FA-solubilized pellet fraction of hippocampal tissue by western blotting with the Tau-5 antibody to detect total tau, the PHF13 antibody to detect Ser396 phosphorylated tau, and the AT8 antibody to detect Ser202/Thr205 phosphorylated tau; β-actin was used as a loading control. *N =* 3 for each genotype group. **(B,D,F,H)** Semi-quantitative densitometric analysis of western blot data determined the ratio of phosphorylated tau to total tau (normalized relative to β-actin). Points indicate measurements from individual mice, bars represent mean values and error bars indicate ± 1 SEM. No significant differences were seen between the groups at 3 months **(B,D)** or for PHF13 at 8-months (**F**; one-way ANOVA). There was a significant difference in phospho-tau levels detected by AT8 in one-way ANOVA (*p <* 0.01) at 8 months **(H)** with *post hoc* multiple comparisons Bonferroni indicating which groups differ; asterisks indicate *p <* 0.05.

In addition to amyloid plaques, another key histopathological hallmark of AD is the development of neurofibrillary tangles containing a hyperphosphorylated form of tau, a microtubule-associated protein (Aillaud and Funke, 2023). Exposure to Aβ induces phosphorylation of tau at Ser396 (Johansson et al., 2006), mediated by glycogen synthase kinase 3 beta (Li and Paudel, 2006). Hyperphosphorylation of Ser396 has been implicated in AD and contributes to reduced microtubule binding (Bramblett et al., 1993). The PHF13 antibody was used to recognize tau phosphorylated at Ser396, while the AT8 antibody recognized tau phosphorylated at Ser202/Thr205 (Li et al., 2017). At 3 months of age, levels of Tau phosphorylation at both Ser396 and Ser202/Thr205 were constant across all 4 genotypes (Figures 2A–D). At 8 months of age, there was a trend (*p* = 0.07) toward increased Ser396 phosphorylation (Figures 2E,F) and a significant increase (*p <* 0.05) in the proportion of Ser202/Thr205 phosphorylation in both the *APOE4* and DM mice compared to WT mice (Figures 2G,H).

In summary, the presence of *APOE4* does not affect levels of APP in the hippocampus at ages up to 8 months. In contrast, *APOE4* does mildly increase tau phosphorylation at age 8 months, though not at 3 months.

### *APOE4* does not increase plaque density in double-mutant mice

**Figure 3:**
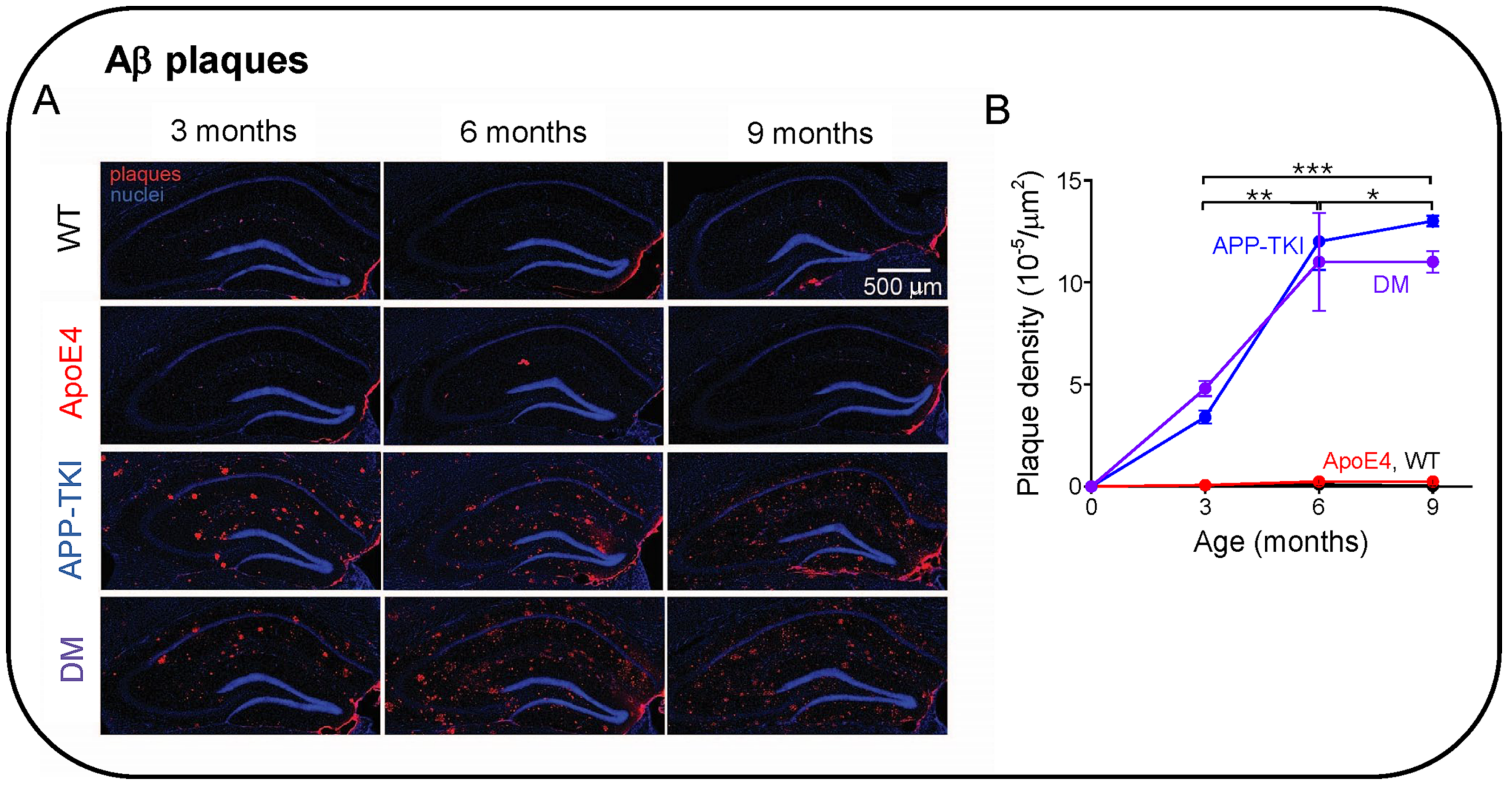
Aβ plaque formation in the hippocampus of Alzheimer’s disease animal models. **(A)** Immunohistochemistry of Aβ pathology in the hippocampus of 3-9-month-old mice. **(B)** Time course of changes in Aβ plaque density in the indicated genotypes. Points represent means and error bars are ± 1 SEM. There were statistically significant interactions between the effects of genotype and age (*N =* 36 mice, two-way ANOVA). Asterisks indicate significant effects of age: *p < 0.05, **p < 0.01, ***p < 0.001. ANOVA with Tukey HSD indicated significant effects of APP-TKI compared to WT or *ApoE4* (*p <* 0.001) and for DM compared to WT or *ApoE4* (*p <* 0.001).

Given that *APOE4* affects Aβ clearance (Verghese et al., 2013; Kanekiyo et al., 2014), we next determined whether *APOE4* influences the accumulation of Aβ plaques in the hippocampus. In these experiments, the amount of fibrillary Aβ plaques was measured in the hippocampus of the four mouse lines at three different ages (3, 6 and 9 months). Plaques were imaged via immunohistochemistry, using an antibody that detects humanized amyloid beta (6E10; Pirttilä et al., 1994). Starting at 3 months of age, both APP-TKI and DM mice had significantly higher amounts of Aβ plaques than WT and ApoE4 mice (Figure 3A). In addition, as the mice aged, there was a dramatic increase in the level of plaques in APP-TKI and DM mice (Figure 3A). The increase in plaque burden, measured as the density of plaques, more than doubled from 3 months to 6 months in APP-TKI and DM mice (Figure 3B). There were significant interaction effects between age and genotype [*F*(6,24) = 9.3, *p <* 0.001]. However, the addition of *APOE4* to the mutant *App* background did not exacerbate formation of Aβ plaques in the hippocampus, consistent with the observed lack of effect of *APOE4* on APP levels (Figure 1). There was also no difference in Aβ42 levels between 3-month-old APP-TKI and DM mice (Supplementary Figure S1).

### Upregulation of vasculature-related genes produced by *APOE4/App* interactions

**Figure 4:**
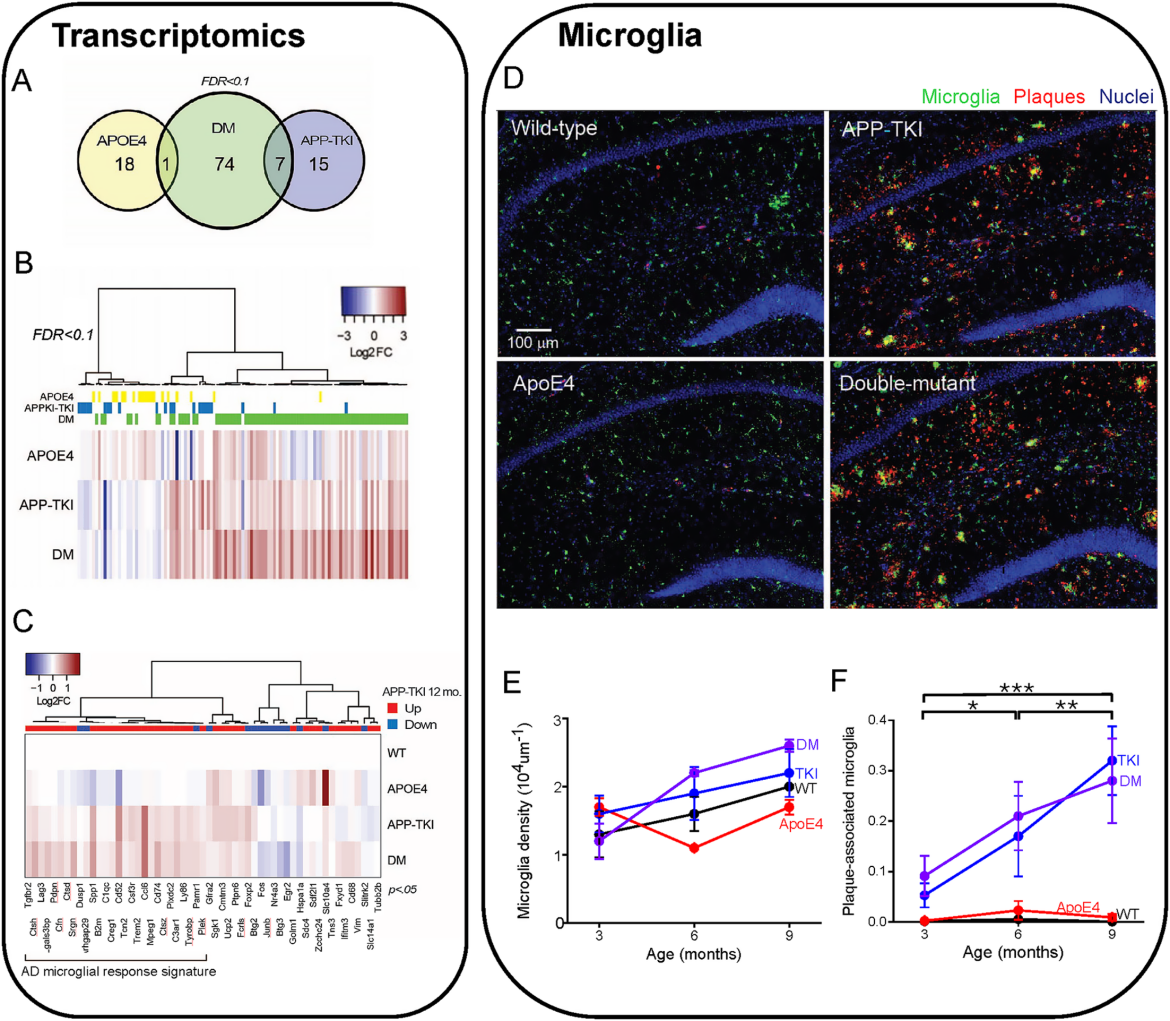
Transcriptomic comparison of 4 mouse lines. **(A)** Venn diagram showing total number and overlapping DEGs in 3-month-old female mice of different genotypes as compared to wild-type (WT) (false discovery rate (FDR < 0.1). **(B)** Heat map of changes in DEGs (log2 fold, or Log2 FCs) compared to WT in each condition (115 genes), sorted via hierarchical clustering (*N =* 3–6 mice for each genotype). DEGs for each mouse genotype are indicated above the heat map. **(C)** DEGs in APP-TKI mice at 12 months, taken from Castillo et al. (2017), that overlap with genes that are significantly different (*p <* 0.05) from WT in any of the three genotypes in our dataset (55 genes). The direction of differential expression for each gene at 12 months, compared to WT, is indicated above the heat map. A large cluster of upregulated genes are mainly associated with microglial activation. **(D)** Hippocampal sections from 4 mouse lines (9 months old) showing immunohistochemical staining for microglia (IBA-1 antibody, green), Aβ plaques (6E10 antibody, red) and nuclei (Hoechst dye, blue). **(E)** Relationship between microglial density and age for each mouse line. There were no statistically significant effects of age or genotype. **(F)** Changes in the fraction of plaque-associated microglia during aging for each mouse line. Points represent mean values and error bars are ± 1 SEM. There was a statistically significant interaction between the effects of genotype and age on the proportion of microglia overlapping with Aβ immunoreactivity (*n =* 36 mice, two-way ANOVA). Asterisks indicate significant effects of age: *p < 0.05, **p < 0.01, ***p < 0.001. ANOVA with Tukey HSD indicated significant effects of APP-TKI compared to WT or *ApoE4* (*p <* 0.001) and for DM compared to WT or *ApoE4* (*p <* 0.001).

Despite the lack of effect of *APOE4* on levels of APP, Aβ or plaques, we next considered the potential consequences of *APOE4/App* interactions on the expression of other genes. For this purpose, we analyzed differentially expressed genes (DEGs) via bulk RNA sequencing of the hippocampus of 3-month-old mice of each mutant genotype and compared these to WT mice. Compared to WT mice, there were 19 DEGs in ApoE4 mice, 22 DEGs in APP-TKI mice and 82 DEGs in DM mice (FDR < 0.1; Figures 4A,B; Supplementary Figure S2). This excludes 8 DEGs that are altered in mouse brain during the estrus cycle (e.g., prolactin and growth hormone; DiCarlo et al., 2017). Only one DEG was shared between ApoE4 and DM mice, and 7 between APP-TKI and DM mice. Gene Ontology analysis of the DEGs showed enrichment in several biological processes, including extracellular matrix organization, for both APP-TKI and DM mice (APP-TKI: 3 genes, *p* = 5.6×10^−3^; DM: 8 genes, *p <* 10^−4^; see Supplementary Table S1). Notably, comparing DM to WT revealed significant enrichment in processes related to the vascular system, including ‘regulation of plasminogen activation’ (3 genes, *p* = 1.3 × 10^−5^) and ‘blood vessel development’ (9 genes, *p* = 9.6 ×10^−3^) that were absent in the single-mutant comparisons. Overall, the low number of DEGs in ApoE4 and APP-TKI indicates that, at 3 months of age, there are only a few select changes in the transcriptomes of the single-mutant mice relative to WTs. In contrast, in the DM mice there were a number of DEGs enriched for distinct biological processes, indicating that the interaction of *ApoE4* and *App* mutations have specific consequences upon gene expression in the brain.

In multiple mouse models of AD, there is a robust activation of genes associated with inflammatory responses and immunological diseases (Castillo et al., 2017; Navakkode et al., 2021; Weissmann et al., 2016). Such transcriptomic changes have been observed in 12-month-old APP-TKI mice (Castillo et al., 2017), so we asked whether these gene expression signatures could also be detected in APP-TKI and DM mice as early as 3 months of age. Because such changes are subtle, due to brain pathology still developing at 3 months (e.g., Figure 3B), we removed the FDR correction and compared the outputs with an *a priori* gene list taken from 12-month-old APP-TKI mice (Castillo et al., 2017). This approach, while increasing the possibility of false positives, allowed us to identify DEGs common to both datasets. The greatest number of shared DEGs were detected in DM mice (DM: 37 genes; APP-TKI: 21 genes and *APOE4*: 15 genes), with many of the genes upregulated in both DM and APP-TKI mice (e.g., *Trem2, Tyrobp, Spp1, Ccl6*) associated with microglial activation and disease (Figure 4C; Keren-Shaul et al., 2017).

Consistent with this inflammation-associated microglial gene signature, we observed an increase in the number of plaque-associated microglia in the hippocampus of APP-TKI and DM mice. Microglia were identified by their expression of ionized calcium-binding adapter-1 (Iba-1; Walker and Lue, 2015) and were evident in the hippocampus in all 4 mouse lines (Figure 4D). Although the density of microglia was not statistically different across all genotypes and age groups (Figure 4E), the proportion of microglia that were associated with Aβ plaques was significantly higher in APP-TKI and DM mice compared to WT and *ApoE4* mutant mice (Figure 4F). This increased prevalence of Iba1-positive, plaque-associated microglia indicates the emergence of disease-associated microglia that could be detected at 3 months of age and continued to increase with age in both APP-TKI and DM mice, but not in WT or *ApoE4* mice (Figure 4F). There were significant interaction effects for age and genotype (*F*(6,24) = 7.3, *p <* 0.001). Thus, while microglial density was similar in all 4 genotypes, the mutant APP gene (and resultant accumulation of Aβ plaques) increased the proportion of microglia associated with plaques. This could account for the observed changes in early inflammation-related microglial gene expression that is likely due to changes in resident microglia rather than accumulation of infiltrating microglia recruited from other brain regions (Sarlus and Heneka, 2017; Sevenich, 2018).

**Figure 5:**
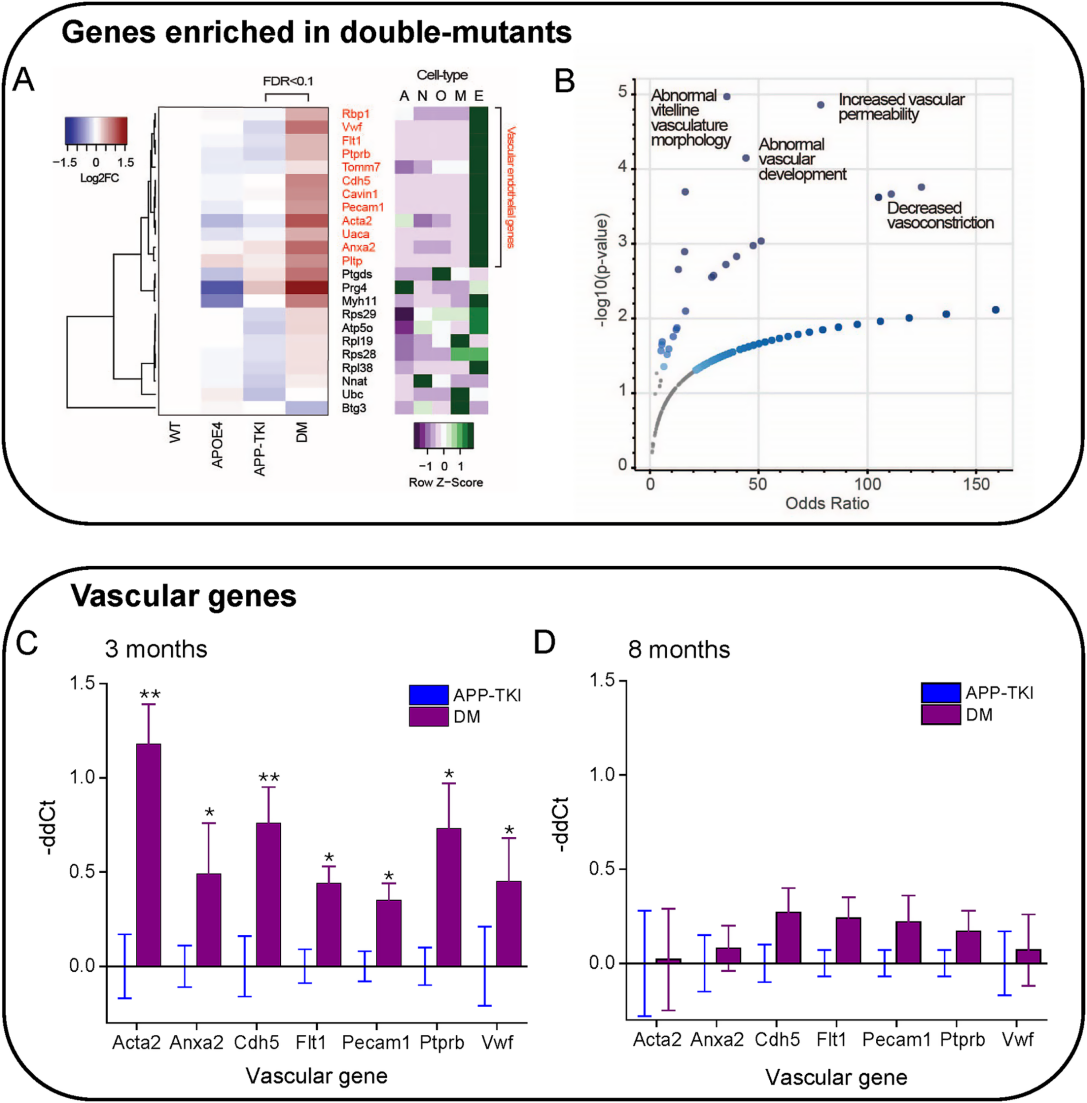
Enrichment of vascular genes in DM mice. **(A)** Hierarchical clustering of genes differentially expressed in APP-TKI compared to DM mice (FDR < 0.1; 23 genes). Right panel shows degree of expression of each gene in major cell types, from the database of Zhang et al. (2014). A: Astrocyte; N: Neuron; O: Oligodendrocyte lineage; M: Microglia; E. Endothelial. A cluster of upregulated genes in DM are enriched in vascular endothelial cells. Z-scores were calculated for each gene from expression values measured in fragments per kilobase of exon per million mapped fragments (FPKM; 12 genes). **(B)** Odds ratios for genes enriched in DM (vs. APP-TKI) in the MGI Mammalian Phenotype Database. Significant terms (*p*-value < 0.05) are indicated by large blue points and selected top terms that are associated with vascular abnormalities are labeled. **(C,D)** Comparison of relative mRNA transcription (-ddCt) for the indicated vascular genes in DM mice compared to APP-TKI mice. **(C)** comparison at 3 months and **(D)** at 8 months. Bars represent means; error bars indicate ± 1 SEM. For each genotype, samples were taken from the hippocampus of 7–8 mice of both sexes. Asterisks indicate significant differences, determined from Wilcoxon signed-rank test, between DM (purple) and APP-TKI (blue): *p < 0.05, **p < 0.01.

To further examine how the interaction of *ApoE4* and mutant *APP* alters gene expression, we analysed differential gene expression between the APP-TKI and DM transcriptomes. This analysis revealed 23 DEGs, all but one of which were more highly expressed in DM compared to APP-TKI (or to ApoE4; Figure 5A; Supplementary Figure S2). Remarkably, most of these DEGs are associated with either vasculature (e.g., *Acta2*, *Ptprb*, *Cdh5, Anxa2, Cavin1, Vwf, Pecam1, Flt1*) or ribosomes (e.g., *Rps29, Rpl38, Rpl19, Rps28*), with the vascular genes the most strongly upregulated (red labels in Figure 5A). In regard to their cellular expression, Figure 5A (right panel) shows that a majority of these vascular genes, as well as a subset of ribosomal genes, are strongly enriched in brain endothelial cells, as compared to neurons (N), astrocytes (A), oligodendrocytes (O) or microglia (M). Comparison of the DEGs enriched in DM mice to the MGI Mammalian Phenotype database (Figure 5B) indicated that most of these DM-associated DEGs are implicated in multiple vascular abnormalities (Zhang et al., 2014). This further establishes the vascular impact of the interaction of *ApoE4* with *APP* mutations.

We next examined the time course of vascular gene upregulation by performing quantitative PCR measurements on RNA from the hippocampus of APP-TKI and DM mice at 3 and 8 months. We focused on the seven most highly upregulated vascular genes detected by our transcriptomic analysis: *Acta2*, *Cdh5*, *Flt1*, *Anxa2*, *Pecam1*, *Ptprb* and *Vwf*. At 3 months of age, transcription levels of all these genes were significantly elevated in DM mice relative to APP-TKI mice, consistent with our RNA sequencing results (Figure 5C). For all except *Acta2*, the increases were not observed in *ApoE4* mice in comparison to WT mice (Supplementary Figure S3), indicating that they arose from interactions between *ApoE4* and the *APP* mutations. Remarkably, in the 8-month-old mice there were no significant differences in levels of expression of these genes between APP-TKI and DM genotypes (Figure 5D).

In summary, our transcriptomic and PCR analyses revealed that several vascular genes were robustly upregulated in DM mice compared to APP-TKI mice, indicating an interaction between *App* and *APOE4*. While these differences were apparent in 3-month-old mice, they were surprisingly absent by 8 months of age. Thus, the changes in vascular gene expression suggest a transient compensatory mechanism that responds to the chronic presence of *APOE4* and the *App* mutations.

### *App* mutations and *APOE4* influence vascular structure

**Figure 6:**
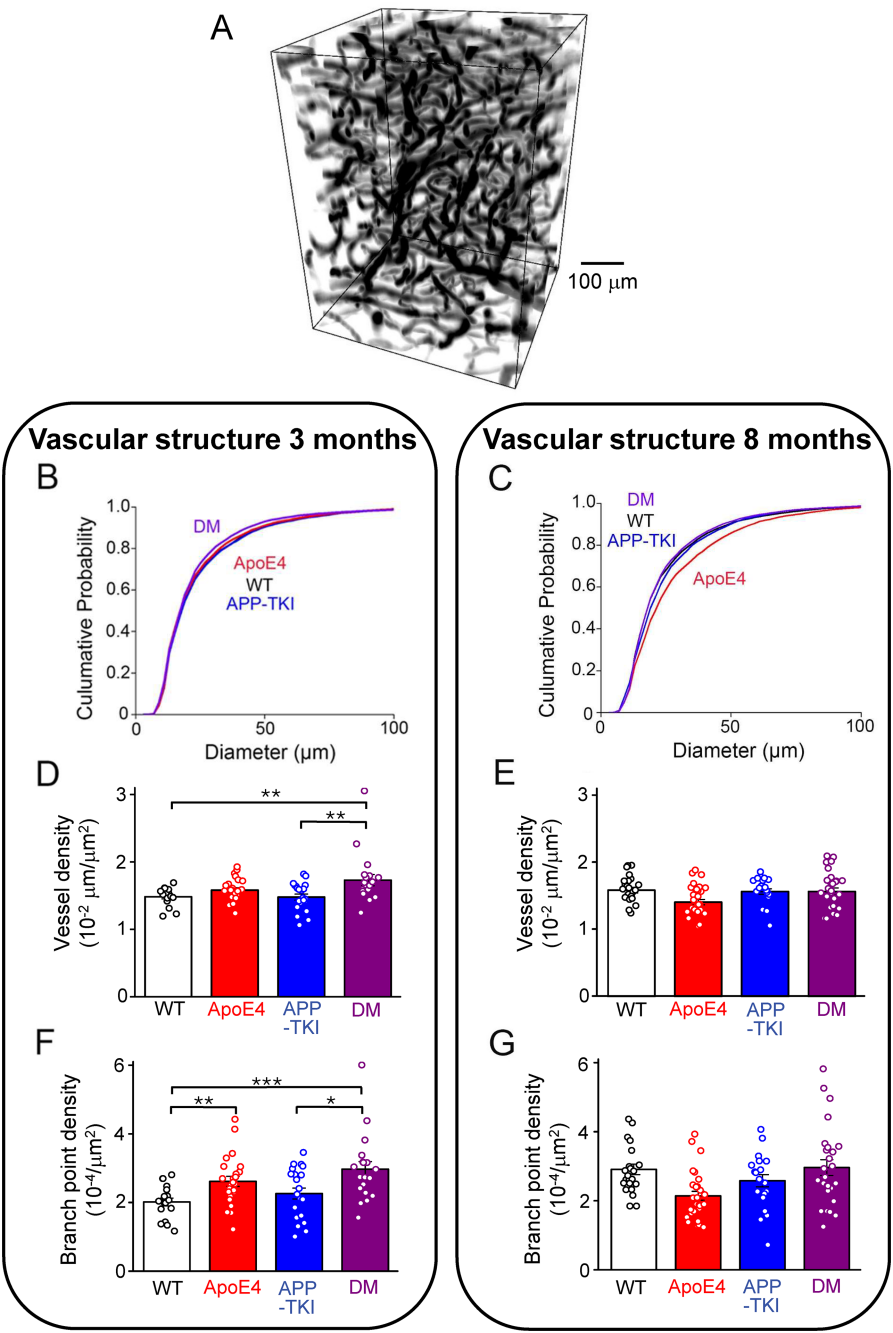
Vascular structure changes in double-mutant mice. **(A)** Representative 3D rendering of a volume of superficial blood vessels in the cortex, obtained via two-photon imaging. **(B)** Cumulative distributions of blood vessel diameters in 3-month-old mice of indicated genotypes. Kruskal-Wallis test followed by Conover’s test revealed significant differences between the following mice: WT vs. DM, *p* = 0.03; APP-TKI vs. *ApoE4*, *p* = 0.01; APP-TKI vs. DM, *p <* 0.0001. There were no differences between WT and *ApoE4* mice (*p* = 0.3), WT and APP-TKI (*p* = 0.1), and *ApoE4* and DM (p = 0.1). **(C)** Cumulative distributions of blood vessel diameters in cortex of 8 month old mice. Kruskal-Wallis test followed by Conover’s test indicated significant differences between the following: WT vs. APP-TKI, p = 0.01; WT vs. *ApoE4*, *p <* 0.0001; APP-TKI vs. ApoE4, *p <* 0.0001; APP-TKI vs. DM, *p* = 9.6 × 10^−4^; and ApoE4 vs. DM: *p <* 0.0001. There were no differences between WT and DM mice (*p* = 0.5). **(D)** Density of blood vessels in 3-month-old mice, determined by ratio of vessel length divided by image area. Statistical analysis (Kruskal-Wallis test, followed by Conover’stest) indicated that vessel density was significantly different between DM and both WT and APP-TKI mice. **(E)** Density of blood vessels in 8-month-old mice; statistical analysis indicated that vessel density was not significantly different between any genotypes. **(F)** Vessel branch point density, normalized by area imaged, for 3-month-old mice. Statistical analysis (Kruskal-Wallis test, followed by Conover’s test) indicated significant differences between several mouse lines. **(G)** Branch point density for 8-month-old mice; statistical analysis indicated significant difference between ApoE4 mice and both WT and DM mice. Bars in D-G represent mean values and error bars show ± 1 SEM. Sample sizes: 3 month old mice - *n =* 27 volumes from 4 WT mice, *n =* 31 samples from 5 APP-TKI mice, *n =* 31 from 4 ApoE4 mice and *n =* 20 from 3 DM mice; 8 month old mice - *n =* 22 from 3 WT mice, *n =* 21 from 3 APP-TKI mice, *n =* 30 from 4 ApoE4 mice and *n =* 27 from 4 DM mice. Significant differences are indicated by asterisks: *p < 0.05; **p < 0.02; ***p < 0.001.

Given the changes in endothelial gene expression observed in the DM mice, we next examined their vascular structure and function. This was done by using *in vivo* 2-photon fluorescence imaging to visualize blood vessels in live mice with fluorescently-labeled dextran injected into their circulatory systems (Figure 6A). Even with the relatively high depth penetration of 2-photon imaging, hippocampal vasculature is too deep to be imaged without damaging the blood vessels lying above the hippocampus. For this reason, we imaged blood vessels in the more superficial cortex.

We first considered blood vessel structure and found that cerebral blood vessels were smaller in diameter in DM mice than in the other 3 genotypes at 3 months of age (Figure 6B). This reduction in DM mice was statistically significant compared to WT and APP-TKI (Kruskal-Wallis test, post-hoc Conover’s test: WT vs. DM, *p* = 0.03; APP-TKI vs. DM, *p <* 10^−4^) and was particularly evident for vessels between 20–80 μm in diameter. In contrast, the diameter of APP-TKI vessels was larger than *ApoE4* (Kruskal-Wallis test, post-hoc Conover’s test *p* = 0.01). Thus, as was observed for the vascular endothelial transcripts, there were differences in vascular structure between DM and APP-TKI mice at 3 months of age. In older mice (8 months old), the mean diameter of blood vessels increased in all 4 genotypes (Figures 6C; Supplementary Figure S3). This age-related growth in vessel diameter has been observed previously (Li et al., 2018; Lowerison et al., 2022). However, there was no difference between the mean diameters of blood vessels in WT and DM brains in 8-month-old mice. This parallels the transient changes in endothelial gene expression that also disappeared by 8 months. However, at this age, blood vessels of DM mice were still smaller than those of APP-TKI mice; vessel diameter was larger in APP-TKI mice than in either DM or WT mice (Kruskal-Wallis test, post-hoc Conover’s test *p <* 0.001 & *p* = 0.01). The most striking change in vascular structure at 8 months was found in *ApoE4* mice, whose vessels were much larger in diameter compared to the other 3 genotypes (Kruskal-Wallis test, post-hoc Conover’s test *p <* 10^−4^; Figure 6C). Thus, while both the *App* mutations and *APOE4* alone increase vessel diameter, their interaction in DM mice prevents this structural action.

Genotype-dependent and age-dependent changes were observed in other features of cerebrovascular structure in DM mice. For example, the density of blood vessels was higher in DM mice at 3 months (Figure 6D; Kruskal-Wallis test, p = 0.01, post-hoc Conover’s test, WT vs. DM: p = 0.01; APP-TKI vs. DM: *p* = 0.02), a difference that disappeared at 8 months (Figure 6E; post-hoc Conover’s test, WT vs. DM: *p* > 0.9999; APP-TKI vs. DM: p > 0.9999). The density of vessel branch points was also higher in DM at 3 months (Figure 6F; Kruskal-Wallis test, *p* = 0.001, post-hoc Conover’s test, WT vs. DM: *p* = 6 × 10^−4^; APP-TKI vs. DM: *p* = 0.04) but this difference was similarly lost in older mice (Figure 6G; post-hoc Conover’s test, WT vs. DM: *p* = 0.9; APP-TKI vs. DM: p = 0.9). The density of branch points was reduced in ApoE4 mice at the older age (Figure 6G; Kruskal-Wallis test, *p* = 0.0003, post-hoc Conover’s test, WT vs. ApoE4: p = 0.001; DM vs. ApoE4: *p* = 0.005), echoing the increase in vessel diameter observed in these mice at this age (Figure 6C).

In summary, at 3 months of age vascular structure differed between DM and APP-TKI mice. Most of these differences were lost in older mice, paralleling the transient transcriptional changes observed in vascular endothelium. In contrast, ApoE4 mice exhibited a pronounced structural phenotype that was only evident in 8-month-old mice and did not track expression of the vascular genes we considered. Thus, the double-mutant mice reveal that the combination of *App* mutations and *APOE4* interact synergistically to influence vascular structure at young age, while at older ages *App* mutations and *APOE4* antagonize each other.

### *App* mutations and *APOE4* influence vascular permeability

**Figure 7:**
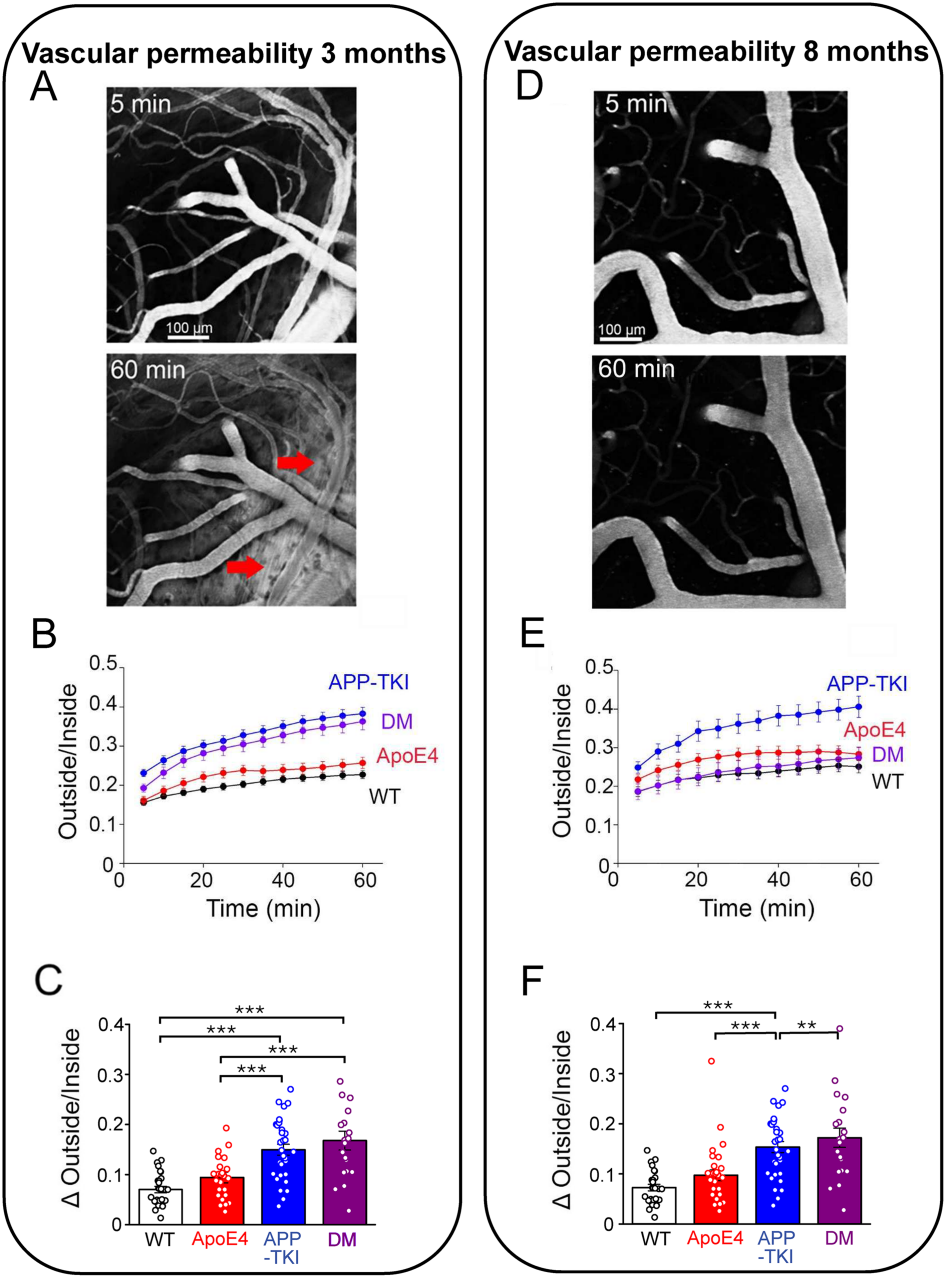
Cerebrovascular permeability measured by leakage of dextran. **(A)** 2-photon images of fluorescence of FITC-labelled dextran (40 kDa) in cortex of a 3 month old DM mouse. At 5 min (top), dextran was largely restricted to blood vessels, but by 60 min (bottom) it had leaked into the surrounding brain tissue (red arrows). **(B)** Time course of dextran leakage in 3 month old mice, measured as ratio of fluorescence outside/inside blood vessels. This ratio significantly changed (two-way repeated-measures ANOVA, time x genotype: *F*_33,1144_ = 9.028, *p <* 0.0001), as a function of time (*F*_1.749,181.9_ = 236.4, *p <* 0.0001) and genotype (*F*_3,104_ = 22.61, *p <* 0.0001). **(C)** Quantitative comparison of blood–brain barrier leakage in 3 month old mice, calculated as difference between outside/inside ratio measured at 5 and 60 min after injecting dextran. Significant differences (Kruskal-Wallis test, followed by Conover’s test) are indicated by asterisks: **p < 0.02; ***p < 0.001. **(D)** 2-photon images of dextran (40 kDa) fluorescence in cortex of an 8 month old DM mouse. **(E)** Time course of dextran leakage in 8 month old mice. Outside/inside ratio significantly changed (two-way repeated-measures ANOVA, time x genotype: *F*_33,1056_ = 6.176, *p <* 0.0001), as a function of both time (*F*_1.713,164.4_ = 122.8, *p <* 0.0001) and genotype (*F*_3,96_ = 7.293, *p <* 0.0001). **(F)** Quantitative comparison of blood–brain barrier leakage in 8 month old mice, calculated as in **(C)**. Significant differences (Kruskal-Wallis test, Conover’s test) are indicated by asterisks: *p < 0.05; ***p < 0.001. Bars and points represent means, while error bars indicate ± 1 SEM. Sample sizes: 3 month old mice - *n =* 27 samples from 4 WT mice, *n =* 31 samples from 5 APP-TKI mice, *n =* 30 from 4 ApoE4 mice and *n =* 20 from 3 DM mice; 8 month old mice - *n =* 22 from 3 WT mice, *n =* 22 from 3 APP-TKI mice, *n =* 31 from 4 ApoE4 mice and *n =* 25 from 3 DM mice.

We next examined vascular blood–brain barrier permeability by tracking the location of fluorescently-labeled dextran (40 kDa) injected into the circulatory system of anesthesized mice. Over time, this tracer leaked from the cerebral vasculature into the brain, evident as a progressive increase in the fluorescence of the extravascular space (red arrows in Figure 7A, bottom). As is evident in Figure 7A, such leakage from the vasculature was non-uniform, being extensive in some areas and absent in others. To quantify the time course of such leakage, in the face of time-dependent, renal clearance of dextran from the circulatory system (Supplementary Figure S5), we calculated the ratio of tracer fluorescence outside the cerebral vasculature relative to fluorescence inside the vasculature (O/I ratio). This O/I ratio increased over time for all genotypes in 3-month-old mice (two-way repeated-measures ANOVA, genotype: *F*_3,104_ = 22.61, *p <* 0.0001), as shown in Figure 7B. There was a rapid initial rise during the first 5 min after dextran injection, followed by a slower, time-dependent leakage over the next hour. Both the initial rise, as well as the secondary increase in O/I ratio (Figure 7C), were greater in APP-TKI and DM mice in comparison to WT controls or ApoE4 mice (Kruskal-Wallis test, *p <* 0.0001, post-hoc Conover’s test, WT vs. APP-TKI: *p <* 0.0001, WT vs. DM: *p <* 0.0001). This indicates higher blood–brain barrier permeability in 3-month-old mice expressing mutant *App*, independent of *APOE4*.

In 8-month-old mice, both the rapid and progressive rises in extravascular fluorescence were still observed (two-way repeated-measures ANOVA, genotype *F*_3,96_ = 7.293, *p <* 0.0001); see Figure 7E. However, the pattern of blood–brain barrier leakage changed across the 4 genotypes (Figure 7F): in these older animals, only APP-TKI mice exhibited high vascular permeability (Kruskal-Wallis test, *p <* 0.0001, post-hoc Conover’s test, WT vs. APP-TKI: *p* = 1.4 × 10^−4^). Thus, in older DM mice *APOE4* served to counteract the effect of *App* mutations on vascular permeability.

To examine the nature of the vascular permeability pathway involved in dye leakage, we compared dextrans of two different molecular weights (40 kDa and 70 kDa). In 3-month-old mice, the permeability of the larger dextran was generally low (Supplementary Figure S6) and not different across the 4 genotypes (Kruskal-Wallis test, *p* = 0.1). This indicates that the blood–brain barrier is permeable only to molecules 40 kDa or less in molecular weight at this young age. In older mice, the permeability to the larger dextran remained low (Supplementary Figure S6), except for a significantly larger leakage of 70 kDa dextran in APP-TKI mice (Kruskal-Wallis test, *p <* 0.0001, Conover’s test, WT vs. APP-TKI: *p* = 4.6 × 10^–4^). Thus, the change in vascular permeability observed in older APP-TKI mice is associated with, and presumably caused by, changes in the size exclusion of the blood–brain barrier.

In summary, the mutant form of *App* expressed in APP-TKI mice increases the permeability of the blood–brain barrier. At 3 months of age, *APOE4* does not influence this effect, while at the older age *APOE4* reverses the actions of the *App* mutations.

## Discussion

By crossing *APOE4* knock-in mice with *App* triple knock-in mice, we generated a novel DM mouse and used this model to examine the *in vivo* consequences of interactions between *App* and *APOE4*. There were no differences in the levels of the APP in DM mice compared to APP-TKI mice. Similarly, there were no differences in the density of amyloid plaques or microglia associated with these plaques. However, compared to APP-TKI mice, DM mice exhibited increased expression of a number of genes that are associated with vascular endothelial cells. Remarkably, these changes were transitory, being present in young mice (3 months old) but absent by 8 months of age. Similarly, DM mice exhibited a number of structural and permeability changes in brain vasculature that differed from APP-TKI mice and were observed in 3-month-old, but not 8-month-old, mice. We conclude that the interaction between *App* mutations and *APOE4* produces effects on vascular endothelial cells that are evident at a young age but are lost later in life.

### A novel mouse model of dementia

Animal models that recapitulate AD pathology are very important for understanding the molecular mechanisms of AD and for developing therapeutic interventions. At present, there are more than 200 mouse models for AD (ALZFORUM, 2026). In most of these, overexpression results in an inability to differentiate between the effects of additional Aβ and of other excessive APP fragments (Barbero-Camps et al., 2014; Chang and Suh, 2005; Saido and Iwata, 2006; Nicolas and Hassan, 2014; Willem et al., 2015). For this reason, our novel mouse model was based on an *App* knock-in mouse (APP-TKI; Saito et al., 2014; Sasaguri et al., 2017) that does not overexpress APP (Figure 1), yet develops robust amyloid deposits at an early age (Figure 3) and exhibits behavioral and pathological phenotypes that are the hallmarks of AD (Saito et al., 2014; Masuda et al., 2016; Sasaguri et al., 2017; Tan et al., 2023). Thus, compared to other mouse lines that rely on transgenic mice to examine *App/APOE4* interactions (e.g., Van Dooren et al., 2006; Montagne et al., 2021; Balu et al., 2023), our mice are more suitable for examining such interactions. This advantage allowed us to specifically detect the effects of early *App/APOE4* interactions, which were manifest on vascular endothelium and cerebral vasculature. Presumably our DM mice represent a model of familial AD, which is often characterized by mutations in *APP* and/or *PSEN* genes and can sometimes occur in an *APOE4* background (Bloss et al., 2008; Bussy et al., 2019; Talboom et al., 2019; Jia et al., 2020; Almkvist et al., 2022). It would also be valuable to develop knock-in models of sporadic AD pathology and determine the effects of *APOE4* in such mice.

It is important to note that our DM mice were heterozygous for *APOE4,* expressing one copy of human *APOE4* and one copy of mouse *ApoE*. This is a limitation because it complicates interpretation of the phenotypes that we observed. Like human *APOE4*, mouse ApoE can associate with Ab plaques (Liao et al., 2015). However, mouse ApoE lacks the domain interaction that is important for the pathogenicity of human APOE4 (Raffai et al., 2001). Thus, mouse ApoE reportedly more closely resembles the human APOE3 variant which is not considered to be an AD risk factor (Lewandowski et al., 2020). While human *APOE4* heterozygotes exhibit an approximate 3-fold to 5-fold increase in risk of AD, *APOE4* homozygotes have an even higher risk (Corder et al., 1993; Farrer et al., 1997; Mahley and Huang, 2012; Neu et al., 2017; Belloy et al., 2019). Thus, it is likely that DM mice with two copies of the *APOE4* allelle would exhibit larger effects associated with *APOE4*/*App* interactions.

### Lack of influence of *APOE4* on amyloid plaques in DM mice

It is generally thought that *APOE4* enhances amyloid plaque levels in the brain, either by promoting plaque formation (Holtzman, 2001; Liu et al., 2017) or by affecting clearance of Aβ from the brain (Verghese et al., 2013; Kanekiyo et al., 2014). Thus, it was surprising that we found no effect of *APOE4* on levels of APP, Aβ or Aβ plaques in DM mice in comparison to APP-TKI mice. This indicates that interactions between *APOE4* and *App* did not influence plaques under our conditions. It is possible that the presence of only one *APOE4* allele in our DM mice yielded *APOE4* levels too low to influence Ab plaques. However, because we did observe numerous consequences of *APOE4* expression on cerebral vasculature in the DM mice, it appears that *APOE4* levels in these mice are sufficient for at least some forms of biological activity. Our work parallels another study that used a mouse model based on mating *APOE4* knock-in mice with 5xFAD transgenic mice that overexpress mutant *App* and *Psen1*. At age 18 months, these mice exhibited no increase in Aβ42 levels compared to controls, aside from a mild increase in the cortex of female mice (Montagne et al., 2021). These results were confirmed in a separate study using a different mouse model based on mating 5xFAD mice with *APOE4* targeted replacement mice: in these mice, increases in Aβ and amyloid plaques were again detected only in older female mice (Balu et al., 2023). Thus, several independent lines of evidence support the idea that the interaction of *APOE4* and *App/Psen1* does not necessarily affect Aβ levels in the mouse brain.

### *APOE4/App* interactions have minimal effects on microglia or tau

Our finding that microglial density is similar in APP-TKI and DM mice indicates that interactions between *APOE4* and *App* also do not influence microglia levels. DM mice also had levels of microglial association with amyloid plaques and activation of microglial response genes that were comparable to those seen in APP-TKI mice. This is similar to what is observed in the 5xFAD/*APOE4* targeted replacement mouse model at a comparable age (Balu et al., 2023). Thus, interactions between *APOE4* and *App* appear to have negligible effects on microglia.

We also found that Tau phosphorylation was unaffected at 3 months of age in all the groups. At 8 months of age, there was an increase in the proportion of phosphorylated tau (Ser202/Thr205) both in the APOE4 and DMs compared to the WT, but not in APP-TKI mice. This indicates that *APOE4* modulates phosphorylation of tau, consistent with previous studies showing that neuronal *APOE4* expression enhances phosphorylated tau (Kobayashi et al., 2003; Harris et al., 2004).

### Early *APOE4-App* interactions impact vascular endothelium

Both *APOE4* (Nation et al., 2019; Montagne et al., 2021; Yamazaki et al., 2021; Liu et al., 2022) and mutant *App* (Nortley et al., 2019; Vandenabeele et al., 2021) have been found to influence the cerebrovascular system. However, most of these studies were performed on older subjects, either post-mortem brains from human AD patients or mice aged 6 months or older. Studies in younger AD model mice have been rare, making it notable that we observed minimal vascular effects of either *APOE4* or mutant *App* alone in 3-month-old mice but found numerous cerebrovascular changes in DM mice at this age.

Because of the traditional focus on older subjects, our knowledge of how *APOE4* and *App* interact during the early stages of AD is incomplete. We discovered that in younger mice vascular gene expression, as well as blood vessel structure and function, exhibited a number of emergent changes that differed from those of *APOE4* or mutant *App* alone. Therefore, these changes must result from the interaction of *APOE4* and *App* mutations at a young age. Compared to APP-TKI (or ApoE4) mice, in DM mice there was increased expression of genes associated with either vasculature or ribosomes. Some of these ribosomal genes were also associated with vasculature. Enriched ribosomal gene expression occurs in some brain endothelial cells, suggesting that these cells are more active in protein synthesis (Vanlandewijck et al., 2018). Moreover, isolated capillaries from young APP-TKI mice exhibit increased cytoplasmic ribosomal proteins (Ito et al., 2023). In human AD patients, ribosomal proteins are enriched in purified blood capillaries but not in parenchymal cells (Suzuki et al., 2022). Thus, it is possible that elevated gene expression triggered by the early interaction of *APOE4* and *App* may be related to enhanced protein synthesis in brain vasculature.

The changes in gene expression observed in 3-month-old DM mice were transient and were absent in 8-month-old DM mice. Therefore, interactions between *APOE4* and *App* apparently are an early event in AD progression. One possible cause of such transient gene expression is early vascular endothelial cell remodeling in response to increased expression of mutant Aβ. Consistent with this possibility, AD patients - some carrying *APOE4* alleles - have higher expression of angiogenic genes, including those identified in our study (e.g., *Vwf*, *Flt1*; Sweeney et al., 2018; Lau et al., 2020). Likewise, selective expression of *APOE4* in vascular mural cells impairs cerebrovascular function and initiates gene expression that regulates angiogenesis (Yamazaki et al., 2021). Thus, it is likely that an early interaction between *APOE4* and *App* triggers vascular endothelial cell remodeling much more than *APOE4* or mutant *App* do alone.

We also observed that *APOE4*/*App* interactions are associated with transient changes in the structure and function of cerebral vasculature. These parallel the transient changes in vasculature-associated gene expression, thereby providing a functional context for the transcriptomic changes. Three-month-old DM mice have smaller and more dense blood vessels, as well as a higher density of branch points. While these changes are apparent in young DM mice, they are absent in older mice. In fact, the valence of *APOE4/App* interactions change with age. In young DM mice, the *App* mutations and *APOE4* synergistically interact to influence vascular structure (most notably thinner blood vessels; Figure 6B). In older mice, *App* mutations and *APOE4* counteract each other: *App* mutations neutralize the effects of *APOE4* on vascular structure (Figure 6C), while *ApoE4* counteracts the effects of *App* mutations on vascular permeability (Figure 7E).

Our most novel finding is that while both vascular gene expression and blood vessel permeability were altered in 3-month-old mice, these changes were largely absent in 8-month-old mice. It is unclear why the consequences of such *APOE4*/*App* interactions are transient. One intriguing possibility is that *APOE4*-driven changes in brain vasculature are intended to repair Aβ-mediated cerebrovascular damage via remodeling during the initial stages of AD. The observed upregulation of angiogenic genes along with an increase in vascular density may indicate activation of homeostatic mechanisms in the brain that counteract Aβ-mediated toxicity. While this hypothesis differs from prevailing toxic gain-of-function models for the *APOE4* allele (Montagne et al., 2021; Jackson et al., 2022), it is consistent with recent reports showing that the *APOE4* allele can confer selective advantages to its carriers, including better performance in cognitive tests (Zokaei et al., 2020), better working memory (Lu et al., 2021) and reduced white matter hyperintensity in human early-stage dementia (Vipin et al., 2023).

While our findings reflect basic research done in mouse models, it is possible to speculate on their potential downstream clinical implications. For example, given the transient changes that *APOE4*/*App* interactions produced in cerebral vasculature, it would be valuable to develop more sensitive diagnostic methods to gauge blood vessel integrity (Stringer et al., 2021; Elschot et al., 2023; Anderle et al., 2025) or cerebral blood flow kinetics (Kim et al., 2023; Vu et al., 2024) and apply such methods to *APOE4* carriers at young ages. Similarly, potential therapeutic interventions could either target the early vascular perturbations that we have identified or could augment the compensatory mechanisms that apparently resolve these vascular consequences at older ages.

### Early stages of AD merit more attention

The majority of research on *APOE4*/*App* interactions, as well as the function of these genes individually, has relied on aged human subjects or mice. For example, our work parallels the study by Montagne et al. (2021) concluding that interaction of *APOE4* and *App/Psen1* mutations accelerates breakdown of the blood–brain barrier. However, because their work was done in old mice (18–24 months), they were unable to detect the early consequences of *APOE4*/*App* interactions that we have uncovered. Understanding the early consequences of *APOE4* expression and its interaction with APP, when the pathology first emerges, is made more important because it is known that *APOE4* can alter cholesterol and lipoprotein levels in young animals (Hamanaka et al., 2000) and that *APOE4* targeted replacement mice exhibit spatial and learning and memory deficits as early as two months of age (Rodriguez et al., 2013). It would also be informative to determine whether the early interactions between *APOE4* and *App* that we observed in DM mice alter neural function or behavior.

## Conclusion

We found that interactions between *APOE4* and *App* selectively and transiently alter vascular gene expression, as well as blood vessel structure and permeability, in young AD model mice. Our discovery highlights the importance of understanding the role of *APOE4*/*App* interactions, particularly in vascular remodeling and angiogenesis, during the very early stages of AD.

## Data Availability

The sequencing data presented in the study are deposited in the NCBI GEO repository, accession number GSE242751. GO analysis data are provided in Supplementary Table S1. All other raw data supporting the conclusions of this article will be made available by the authors, without undue reservation.

## Supplementary material

SUPPLEMENTARY FIGURE S1 **(A)** Western blot of humanized Aβ42 protein in the supernatant soluble fraction of hippocampal tissue in 3-month-old mice. Hippocampal samples were ultracentrifuged at 100,000 x g for 1 hour at 4°C and the resulting pellets were resuspended in ice-cold formic acid (100 μl; F0507, Sigma-Aldrich), sonicated with a probe sonicator at 35% amplitude amd neutralized with 2.5M Tris (pH 8.5). The formic acid fraction was probed with a human-specific ꞵ-amyloid antibody (clone D3E10, 12843S, Cell Signaling Technology, 1:1000), with ꞵ-actin was used as an endogenous loading control (A5441, Sigma-Aldrich). **(B)** Comparison of A42 levels, quantified by normalization to β-Actin. Bars represent means and error bars are SEM. N=3 for each genotype. No significant differences were detected between groups in one-way ANOVA.

SUPPLEMENTARY FIGURE S2 **(A–D)** Volcano plots showing log2 fold change compared to -log10 p-values for differential expression tests for all expressed protein-coding genes. Differentially expressed genes (FDR<0.1, excluding 8 genes differentially expressed during the estrus cycle) are indicated in red and the FDR threshold is marked by a dashed line. Selected top DEGs are labeled in each plot.

SUPPLEMENTARY FIGURE S3 Relative mRNA transcription (-ddCt) of 7 vascular genes across the 4 genotypes at **(A)** 3 months and **(B)** 8 months: Acta2 (i), Cdh5 (ii), Flt1 (iii), Anxa2 (iv), Pecam1 (v), Ptprb (vi) and Vwf (vii). 3 months: WT (*n =* 7), APP-TKI (*n =* 7), APOE (*n =* 7) and DM (*n =* 7). 8 months: WT (*n =* 8), APP-TKI (*n =* 8), APOE (*n =* 8) and DM (*n =* 8). Data were shown as means ± SEM, and asterisks denote statistical significance; *p<0.05 and **p<0.005. The statistical test was by one-way ANOVA Brown-Forsythe test followed by a post hoc Dunnett T3 multiple comparison test.

SUPPLEMENTARY FIGURE S4 Comparison of blood vessel diameters at different ages. Two-way ANOVA results: age x genotype: F3,50867 = 29.56, *P <* 0.0001, age: F1,50867 =122.8, *P <* 0.0001, genotype: F3,50867 = 37.27, *P <* 0.0001, Sidak's multiple comparisons test, WT 3 month vs. 7-9 month: *p =* 0.12, APP-TKI 3 month vs. 7-9 month: *p =* 0.051, ApoE4 3 month vs. 7-9 month: *p <* 0.0001, DM 3 month vs. 7-9 month: *p =* 0.0002). Data represent mean ± 1 SEM. (Young, *N =* 4, *n =* 27 WT, N =5, *n =* 31 APP-TKI, *N =* 4, *n =* 31 ApoE4, *N =* 3, *n =* 20 DM; Old, *N =* 3, *n =* 22 WT, N =3, *n =* 21 APP-TKI, *N =* 4, *n =* 30 ApoE4, *N =* 4, *n =* 27 DM).

SUPPLEMENTARY FIGURE S5 Measurement of dextran fluorescence within the circulatory system and surrounding brain tissue of 8-month-old WT mice. **(A)** Time course of fluorescence changes within blood vessels (inside) and in surrounding brain tissue (outside). At all times, fluorescence is much higher within blood vessels than outside the vessels, indicating minimal dextran leakage under these conditions. Nonetheless, fluorescence within the blood vessels declines over time, due to renal clearance of dextran. **(B)** Ratio of the two plots shown in **(A)**, illustrating a relatively constant outside/inside ratio over the course of the measurement period. Points indicate mean values measured over 22 cortical areas, while error bars indicate ± 1 SEM.

SUPPLEMENTARY FIGURE S6 The change of O/I ratio of a larger tracer (70 kDa dextran) at different ages across genotypes. **(A,B)** Difference between the O/I ratio at 5 min and 60 min after injection for young (Kruskal-Wallis test, *p =* 0.1, Conover’s multiple comparisons test, WT vs. APP-TKI: p > 0.9999, WT vs. ApoE4: p > 0.9999, WT vs. DM: *p =* 0.2, APP-TKI vs. ApoE4: *p* > 0.9999, APP-TKI vs. DM: *p =* 0.2, ApoE4 vs. DM: p =0.13) and old (Kruskal-Wallis test, *p <* 0.0001, Conover’s multiple comparisons test, WT vs. APP-TKI: p =4.6 x 10-4, WT vs. ApoE4: *p =* 0.5, WT vs. DM: *p =* 0.5, APP-TKI vs. ApoE4: *p <* 0.0001, APP-TKI vs. DM: *p =* 0.01, ApoE4 vs. DM: *p =* 0.2) ages. Data present mean ± 1 SEM. (Young, *N =* 4, *n =* 27 WT, N =5, *n =* 31 APP-TKI, *N =* 4, *n =* 30 ApoE4, *N =* 3, *n =* 20 DM; Old, *N =* 3, *n =* 22 WT, N =3, *n =* 22 APP-TKI, *N =* 4, *n =* 31 ApoE4, *N =* 4, *n =* 25 DM).

SUPPLEMENTARY TABLE S1 Details of analysis of transcriptomic changes in AD mouse models. Sheet 1: Differential gene expression (DE) in APP-TKI vs. DM vs. WT mice. Sheet 2: Differential gene expression in ApoE4 vs. WT mice. Sheet 3: Gene ontogeny (GO) analysis for genes differentially expressed in ApoE4 mice vs. WT mice. Sheet 4: GO analysis for genes differentially expressed in APP-TKI mice vs. WT mice. Sheet 5: GO analysis for genes differentially expressed in DM mice vs. WT mice. Sheet 6: GO analysis for genes differentially expressed in APP-TKI mice vs. DM mice. Sheet 7: Primer sequences used for qPCR analysis.
