# Supplementary figures and images for: APOE4-APP interactions exert early influences on cerebrovascular structure and function: implications for Alzheimer’s disease
Source: Front Neurosci. 2025 Oct 24;19:1629830. doi: 10.3389/fnins.2025.1629830 (PMC12592981; doi:10.3389/fnins.2025.1629830)

**Figure S1 – Guo et al.**

## Humanized A $\beta$ 42 protein

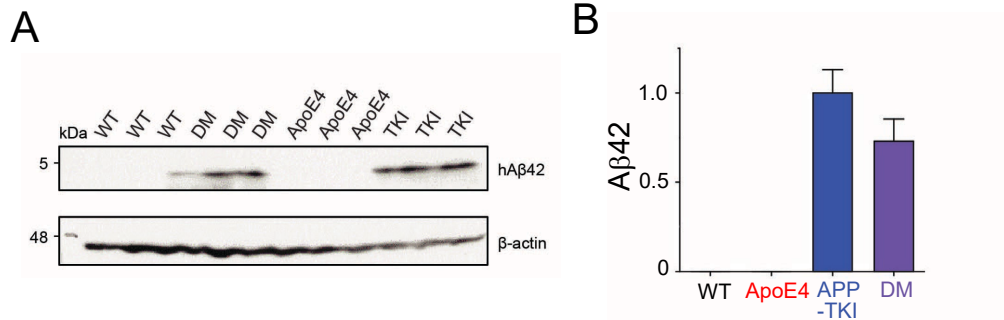

Supplement: SUPPLEMENTARY FIGURE S1 — (A) Western blot of humanized Aβ42 protein in the supernatant soluble fraction of hippocampal tissue in 3-month-old mice. Hippocampal samples were ultracentrifuged at 100,000 x g for 1 hour at 4°C and the resulting pellets were resuspended in ice-cold formic acid (100 μl; F0507, Sigma-Aldrich), sonicated with a probe sonicator at 35% amplitude amd neutralized with 2.5M Tris (pH 8.5). The formic acid fraction was probed with a human-specific ꞵ-amyloid antibody (clone D3E10, 12843S, Cell Signaling Technology, 1:1000), with ꞵ-actin was used as an endogenous loading control (A5441, Sigma-Aldrich). (B) Comparison of A42 levels, quantified by normalization to β-Actin. Bars represent means and error bars are SEM. N=3 for each genotype. No significant differences were detected between groups in one-way ANOVA. [file Image_1.pdf]

**Figure S2 – Guo et al.**

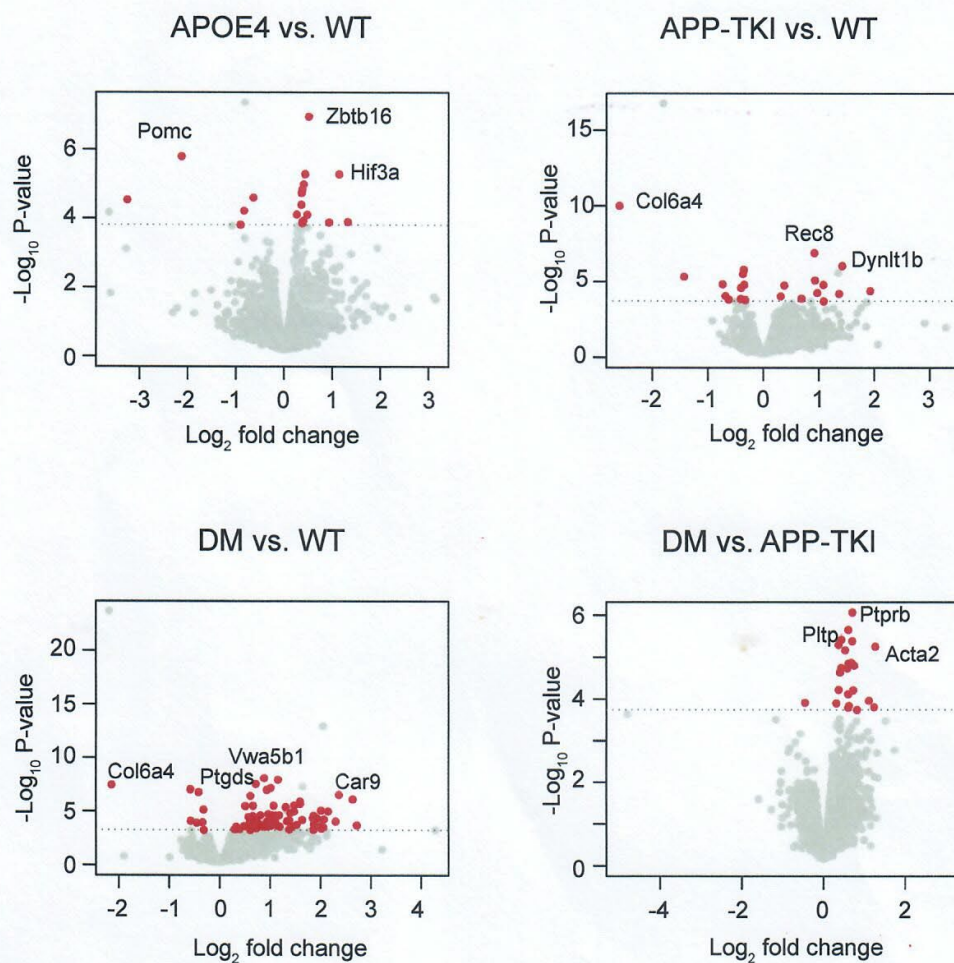

Supplement: SUPPLEMENTARY FIGURE S2 — (A–D) Volcano plots showing log2 fold change compared to -log10 p-values for differential expression tests for all expressed protein-coding genes. Differentially expressed genes (FDR<0.1, excluding 8 genes differentially expressed during the estrus cycle) are indicated in red and the FDR threshold is marked by a dashed line. Selected top DEGs are labeled in each plot. [file Image_2.pdf]

Figure S3 – Guo et al.

A 3 months

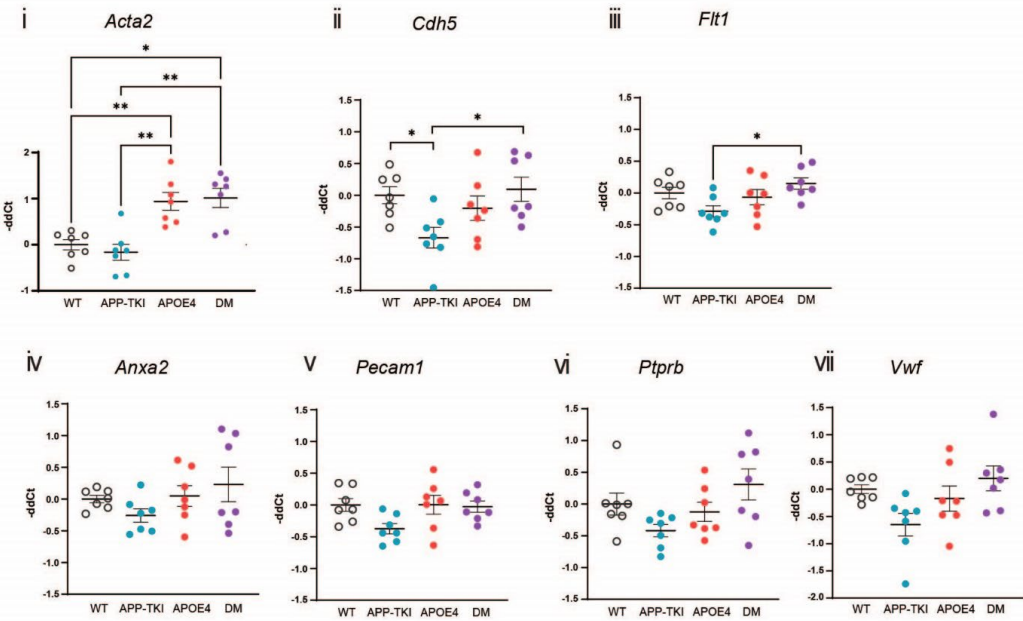

B 8 months

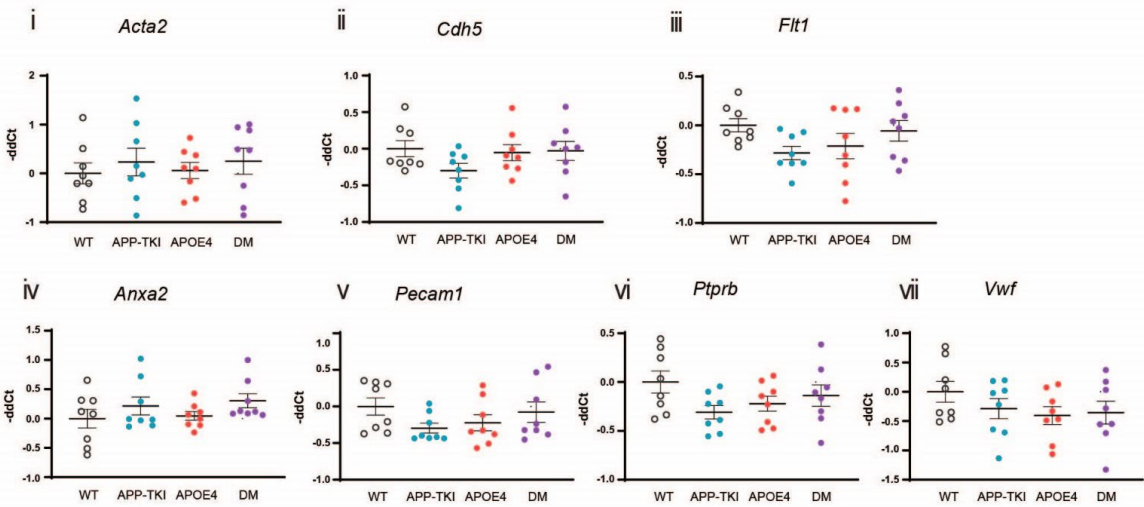

Supplement: SUPPLEMENTARY FIGURE S3 — Relative mRNA transcription (-ddCt) of 7 vascular genes across the 4 genotypes at (A) 3 months and (B) 8 months: Acta2 (i), Cdh5 (ii), Flt1 (iii), Anxa2 (iv), Pecam1 (v), Ptprb (vi) and Vwf (vii). 3 months: WT (n = 7), APP-TKI (n = 7), APOE (n = 7) and DM (n = 7). 8 months: WT (n = 8), APP-TKI (n = 8), APOE (n = 8) and DM (n = 8). Data were shown as means ± SEM, and asterisks denote statistical significance; *p<0.05 and **p<0.005. The statistical test was by one-way ANOVA Brown-Forsythe test followed by a post hoc Dunnett T3 multiple comparison test. [file Image_3.pdf]

Figure S4 – Guo et al.

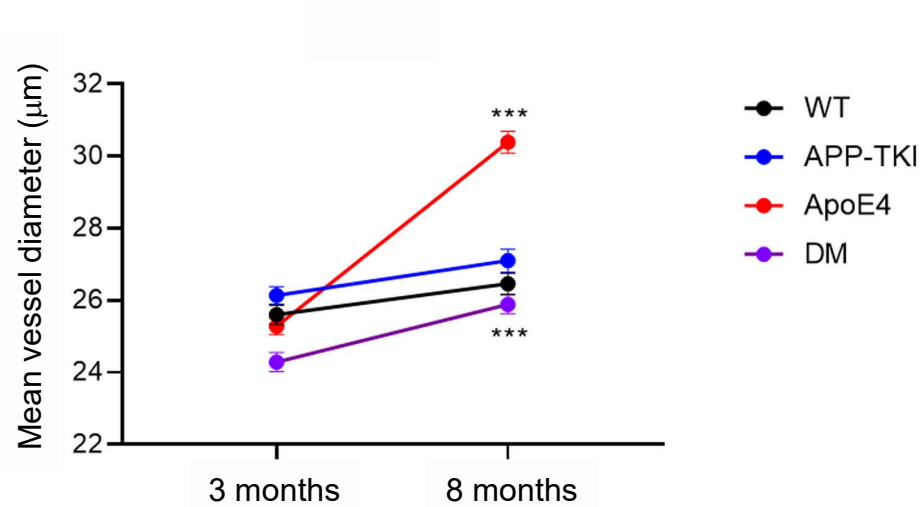

Supplement: SUPPLEMENTARY FIGURE S4 — Comparison of blood vessel diameters at different ages. Two-way ANOVA results: age x genotype: F3,50867 = 29.56, P < 0.0001, age: F1,50867 =122.8, P < 0.0001, genotype: F3,50867 = 37.27, P < 0.0001, Sidak's multiple comparisons test, WT 3 month vs. 7-9 month: p = 0.12, APP-TKI 3 month vs. 7-9 month: p = 0.051, ApoE4 3 month vs. 7-9 month: p < 0.0001, DM 3 month vs. 7-9 month: p = 0.0002). Data represent mean ± 1 SEM. (Young, N = 4, n = 27 WT, N =5, n = 31 APP-TKI, N = 4, n = 31 ApoE4, N = 3, n = 20 DM; Old, N = 3, n = 22 WT, N =3, n = 21 APP-TKI, N = 4, n = 30 ApoE4, N = 4, n = 27 DM). [file Image_4.pdf]

Figure S5 – Guo et al.

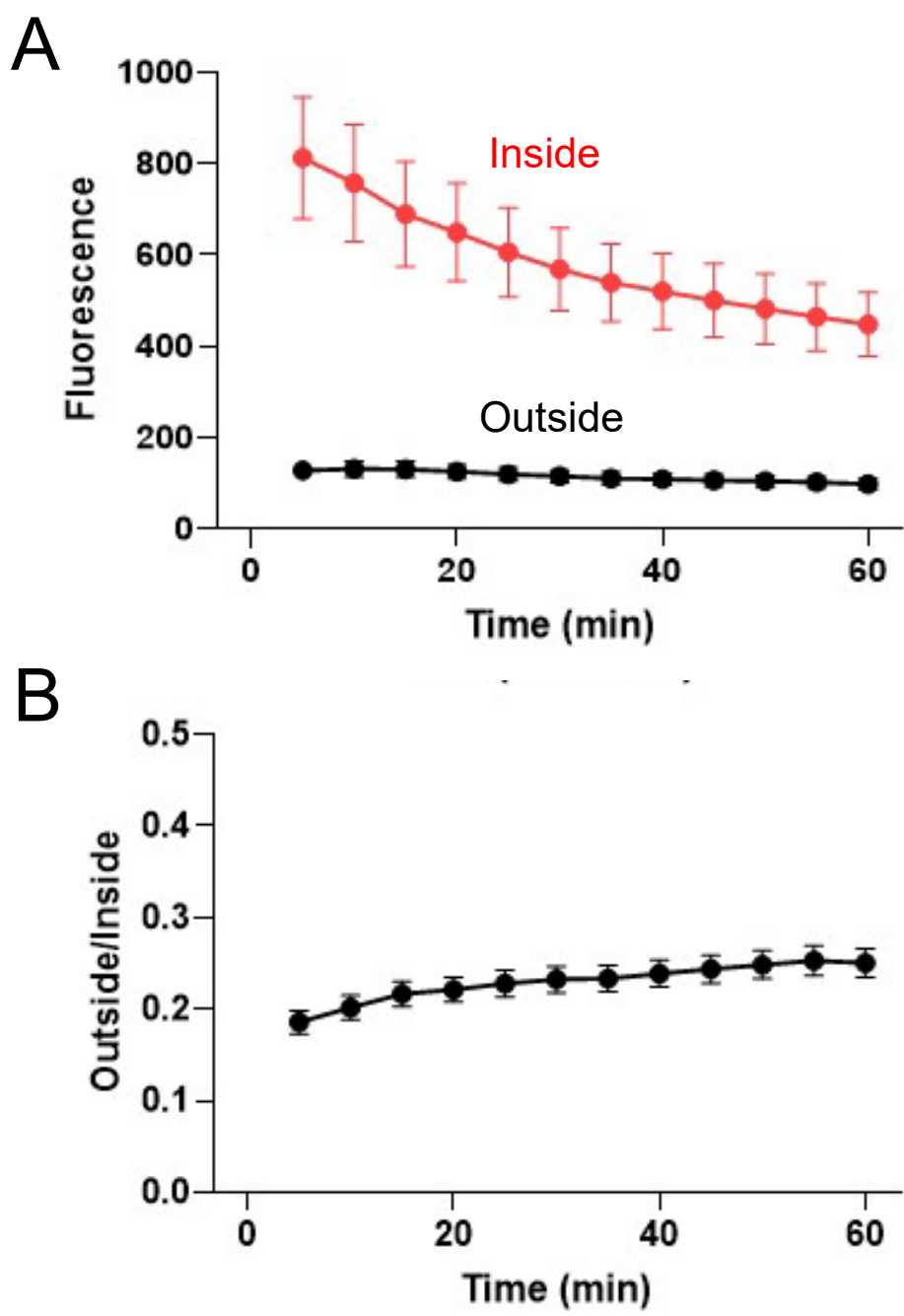

Supplement: SUPPLEMENTARY FIGURE S5 — Measurement of dextran fluorescence within the circulatory system and surrounding brain tissue of 8-month-old WT mice. (A) Time course of fluorescence changes within blood vessels (inside) and in surrounding brain tissue (outside). At all times, fluorescence is much higher within blood vessels than outside the vessels, indicating minimal dextran leakage under these conditions. Nonetheless, fluorescence within the blood vessels declines over time, due to renal clearance of dextran. (B) Ratio of the two plots shown in (A), illustrating a relatively constant outside/inside ratio over the course of the measurement period. Points indicate mean values measured over 22 cortical areas, while error bars indicate ± 1 SEM. [file Image_5.pdf]

Figure S6 – Guo et al.

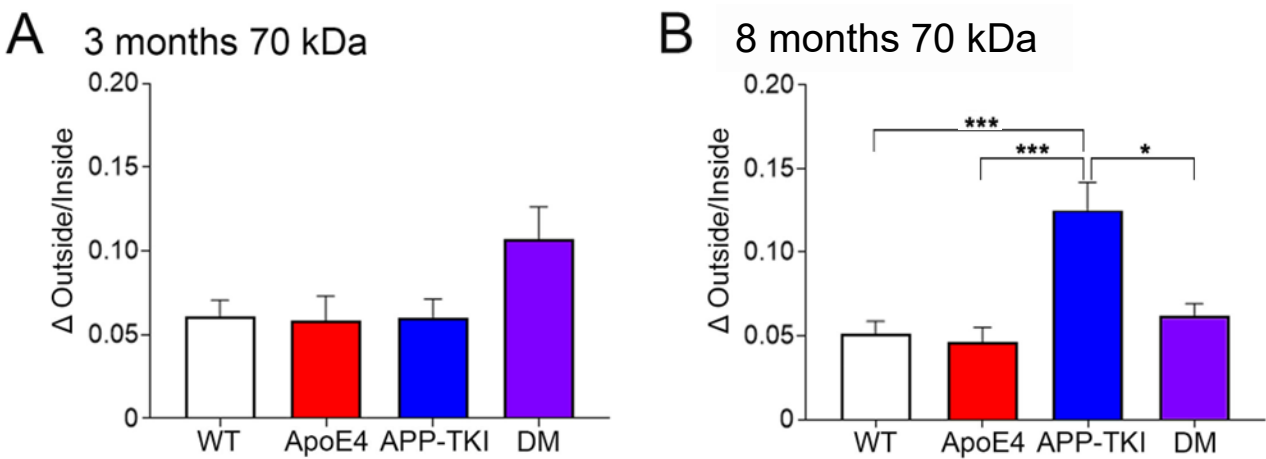

Supplement: SUPPLEMENTARY FIGURE S6 — The change of O/I ratio of a larger tracer (70 kDa dextran) at different ages across genotypes. (A,B) Difference between the O/I ratio at 5 min and 60 min after injection for young (Kruskal-Wallis test, p = 0.1, Conover’s multiple comparisons test, WT vs. APP-TKI: p > 0.9999, WT vs. ApoE4: p > 0.9999, WT vs. DM: p = 0.2, APP-TKI vs. ApoE4: p > 0.9999, APP-TKI vs. DM: p = 0.2, ApoE4 vs. DM: p =0.13) and old (Kruskal-Wallis test, p < 0.0001, Conover’s multiple comparisons test, WT vs. APP-TKI: p =4.6 x 10^-4^, WT vs. ApoE4: p = 0.5, WT vs. DM: p = 0.5, APP-TKI vs. ApoE4: p < 0.0001, APP-TKI vs. DM: p = 0.01, ApoE4 vs. DM: p = 0.2) ages. Data present mean ± 1 SEM. (Young, N = 4, n = 27 WT, N =5, n = 31 APP-TKI, N = 4, n = 30 ApoE4, N = 3, n = 20 DM; Old, N = 3, n = 22 WT, N =3, n = 22 APP-TKI, N = 4, n = 31 ApoE4, N = 4, n = 25 DM). [file Image_6.pdf]
